# The Genetics of Neuropathic Pain from Model Organisms to Clinical Application

**DOI:** 10.1016/j.neuron.2019.09.018

**Published:** 2019-11-20

**Authors:** Margarita Calvo, Alexander J. Davies, Harry L. Hébert, Greg A. Weir, Elissa J. Chesler, Nanna B. Finnerup, Roy C. Levitt, Blair H. Smith, G. Gregory Neely, Michael Costigan, David L. Bennett

**Affiliations:** 1 Departamento de Fisiología, Facultad de Ciencias Biológicas, Pontificia Universidad Católica de Chile, Santiago, Chile; 2 Neural Injury Group, Nuffield Department of Clinical Neuroscience, John Radcliffe Hospital, University of Oxford, Oxford, UK; 3 Chronic Pain Research Group, Division of Population Health and Genomics, Mackenzie Building, Ninewells Hospital & Medical School, University of Dundee, Dundee, UK; 4 The Jackson Laboratory, Bar Harbor, ME, USA; 5 Department of Clinical Medicine, Danish Pain Research Center, Aarhus University, Aarhus 8000, Denmark; 6 Department of Anesthesiology, Perioperative Medicine and Pain Management, and John T. MacDonald Foundation Department of Human Genetics, Miller School of Medicine, University of Miami, Miami, FL, USA; 7 Dr. John and Anne Chong Lab for Functional Genomics, Camperdown, University of Sydney, Sydney, NSW, Australia; 8 Departments of Anesthesia and Neurobiology, Children’s Hospital Boston and Harvard Medical School, Boston, MA, USA

## Abstract

Neuropathic pain (NeuP) arises due to injury of the somatosensory nervous system and is both common and disabling, rendering an urgent need for non-addictive, effective new therapies. Given the high evolutionary conservation of pain, investigative approaches from *Drosophila* mutagenesis to human Mendelian genetics have aided our understanding of the maladaptive plasticity underlying NeuP. Successes include the identification of ion channel variants causing hyper-excitability and the importance of neuro-immune signaling. Recent developments encompass improved sensory phenotyping in animal models and patients, brain imaging, and electrophysiology-based pain biomarkers, the collection of large well-phenotyped population cohorts, neurons derived from patient stem cells, and high-precision CRISPR generated genetic editing. We will discuss how to harness these resources to understand the pathophysiological drivers of NeuP, define its relationship with comorbidities such as anxiety, depression, and sleep disorders, and explore how to apply these findings to the prediction, diagnosis, and treatment of NeuP in the clinic.

## Teaser

Calvo et al. discuss how applying genetic techniques, from model organisms to human populations, can help us understand the pathophysiology of neuropathic pain. These strategies could soon reveal novel analgesic drug targets and aid both personalized risk prediction and treatment.

## Main Text

Neuropathic pain (NeuP) arises as a consequence of a lesion or disease of the somatosensory nervous system (Jensen et al., 2011). It is common, affecting 7%–10% of the general population, and its prevalence is projected to increase with the aging population, diabetes epidemic, and improved cancer survival (van Hecke et al., 2014). Unfortunately, current drug treatments for NeuP are inadequate due to both poor efficacy and tolerability (Finnerup et al., 2015). NeuP is also associated with a high level of disability and a large socio-economic cost: the global burden of disease survey showed that chronic pain is the third most important cause of disability-adjusted life-years worldwide (GBD 2017 Disease and Injury Incidence and Prevalence Collaborators, 2018, Rice et al., 2016). The current opioid epidemic exemplifies the problems associated with long term treatment of NeuP and the need for alternative non-addictive therapeutics (Jones et al., 2018). We do not yet have a full understanding of the drivers of NeuP and the extent to which they depend on the specific underlying etiology. For example, are the factors that precipitate NeuP in traumatic nerve injury and post-herpetic neuralgia the same? Nor do we have a full explanation as to why, following the same insult, some patients develop NeuP and others do not. Why for instance do only 30%–50% of diabetic polyneuropathy patients develop NeuP (Feldman et al., 2017)? Differences here are likely to depend on a complex interaction between environmental and genetic factors that alter both the vulnerability and resilience of the somatosensory nervous system. A better understanding of the genetic architecture of NeuP will therefore provide fundamental insights into disease pathophysiology, help us understand inter-individual variation in NeuP, and reveal new drug targets.

Despite great efforts, the development of new and effective treatments for NeuP has proved difficult, with a number of promising pharmacological targets identified in preclinical studies failing to achieve clinical efficacy (Percie du Sert and Rice, 2014). The reasons for this are complex, spanning the whole process of drug development but broadly falling into two categories: the sensitivity of clinical trials for NeuP therapies due to the subjectivity of pain reporting, the placebo effect and case mix (Finnerup et al., 2018) and the ability of animal models to predict clinical efficacy (Percie du Sert and Rice, 2014).

There have been major advances in gene sequencing technology and the informatics required to deal with large data volumes. These are now being applied at a national scale to health services, for instance, the sequencing of 100,000 whole genomes by the National Health Service (NHS) in the UK (Samuel and Farsides, 2017). The sensory phenotyping of NeuP has become more precise and can now be combined with large clinical and research cohorts such as the UK-Biobank of 500,000 people (Sudlow et al., 2015). The HEAL initiative in the USA (https://heal.nih.gov) is also collecting pain outcome measures and genetic data (Volkow and Koroshetz, 2019) providing opportunities to create large-scale international consortia (Pascal et al., 2019). Complementary advances in mouse genetics have improved the precision of basic research into the discovery of pain mechanisms. The speed with which we can manipulate the genome of model organisms such as the fruit fly and mouse, or even human sensory neurons derived from stem cells, means we can rapidly interrogate gene function once genes of interest are identified. Given the multi-disciplinary expertise needed to make full use of these resources, we organized a joint satellite meeting of the NeuP and Genetics Special Interest Groups (SIGs) of the International Association for the Study of Pain (IASP) at The Jackson Laboratory, Bar Harbor, ME (September 10 and 11, 2018), which inspired many of the ideas presented in this review.

### Assessment of NeuP in Patients

**Figure 1:**
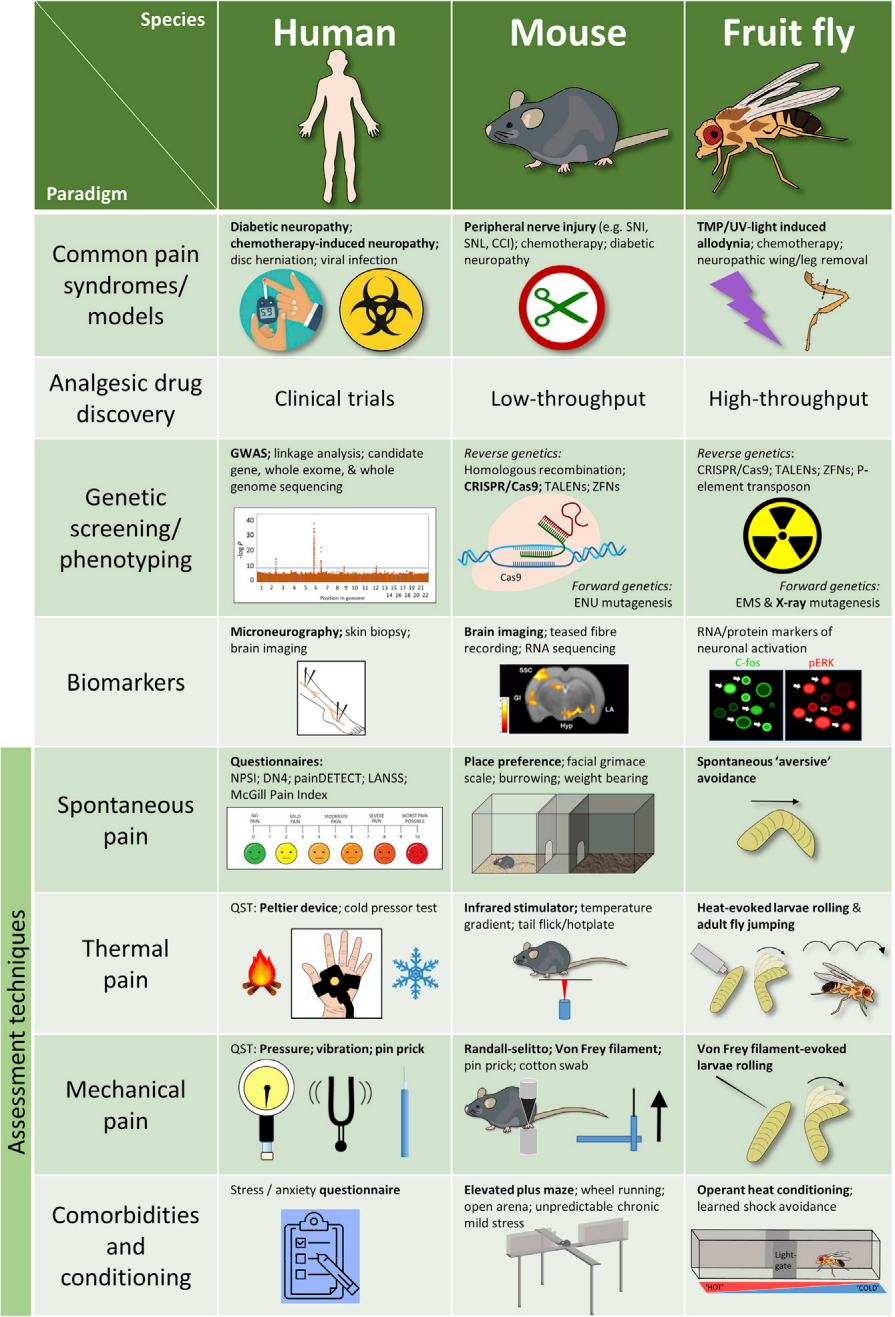
A Comparison of the Genetic Investigation and Functional Analysis of NeuP in Human and Animal Models The study of NeuP in humans has the obvious advantage of verbal feedback of the individual’s subjective experience. The development of relatively non-invasive neurophysiological techniques has allowed us to further probe for diagnostic and functional biomarkers in NeuP patients. The house mouse (*Mus musculus*) and fruit fly (*Drosophila melanogaster*) are genetically modifiable organisms commonly used in studies of nociception and pain processing. High-throughput genetic and phenotypic analysis (e.g., nociception) can be achieved in flies, as well as fish and worms, whereas complex behavioral traits such as cognitive affect can be assessed in rodents. Appreciation of plasticity in the fly nervous system has led to the application of several injury and chemically induced neuropathy models to this organism (Khuong et al., 2019). Adult flies are also capable of learned conditioning to thermal stimuli such that they subsequently avoid that area of the chamber even in the absence of heat (Wustmann et al., 1996). Mutants displaying spontaneous avoidance behavior that mimics the stimulus-elicited avoidance response in wild-type larvae remain to be investigated as a possible measure of ongoing aversive sensation (Heiman et al., 1996). Abbreviations: CCI, chronic constriction injury; CRISPR/Cas9, clustered regularly interspaced palindromic repeats/CRISPR-associated protein-9 nuclease; DN4, Neuropathic Pain Diagnostic Questionnaire; EMS, ethyl methanesulfonate; ENU, N-ethyl-N-nitrosourea; GWAS, whole-genome association study; LANNS, Leeds Assessment of Neuropathic Symptoms and Signs; NPSI, neuropathic pain symptom inventory; QST, quantitative sensory testing; SNI, spared nerve injury; SNL, spinal nerve ligation; TALENs, transcription activator-like effector nucleases); TMP/UV, trimethylpsoralen/ultra violet; ZFN, zinc-finger nuclease.

The past decade has seen great improvements in the techniques used to define the sensory profile of NeuP patients (Figure 1). These include questionnaires to assess pain quality, psychophysical tools to assess sensory perception, and alteration of experimental pain through conditioned pain modulation (CPM). These questionnaires e.g., NPSI, DN4, painDETECT, and LANSS (Bennett et al., 2007) incorporate descriptors of sensory symptoms to generate a score that helps predict whether the pain is likely to be neuropathic or not, and to characterize distinct dimensions of NeuP. Psychophysical tools to test the function of the somatosensory nervous system have benefited from standardization of quantitative sensory testing (QST) protocols. This consistency of reporting enables the generation of large cohorts of patients from different centers, so enhancing statistical power (Rolke et al., 2006). Conditioned pain modulation are dynamic psychophysical protocols that aim to explore an individual’s descending pain modulatory system, although the complexity of the protocols and the variability in the size and stability of the response remains a challenge (Kennedy et al., 2016). Deployment of a combination of the above techniques now enables the stratification of NeuP patients according to sensory profile, providing a much richer dataset than simply classifying patients according to etiology (e.g., polyneuropathy versus post-herpetic neuralgia) and enabling for selection of patients with specific phenotypes to empower clinical trials (Attal et al., 2018). There have also been significant advances in the development of biomarkers which assess neurobiological processes underlying pain but are not dependent on patient report, such as neurophysiology (microneurography and evoked potentials) and neuro-imaging (Tracey et al., 2019). When different biomarkers are used in combination they can become objective indicators with adequate specificity and sensitivity to aid diagnosis and prognosis (Tracey et al., 2019).

Combining such sensory phenotyping and biomarkers with genetics should provide significant added value in understanding the pathophysiology of NeuP and be of clinical relevance in terms of diagnosis, prognosis, and treatment choice. Their use will depend on the cohort being studied. For example, questionnaires can be applied at population level, while QST is only feasible in patients attending specialized services. However, standardization of QST protocols has enabled increased cohort size. These methods have associated gene variants with particular aspects of the NeuP sensory phenotype, such as paradoxical heat sensation (the perception of warmth/heat as the skin is cooled) (Binder et al., 2011). Brain imaging is now also being used at the population level; for instance, UK-Biobank plans to image 100,000 predominantly healthy genome-sequenced individuals, who will then be followed longitudinally. An initial analysis has already shown that structural and functional brain imaging phenotypes are heritable (Elliott et al., 2018), with over 100 genomic regions showing associations with imaging phenotypes. Although such imaging studies are not undertaken using an experimental pain model, clinical pain data *are* being collected, and they do begin to provide insight into the genetic basis of brain structure/connectivity. This will help inform on the brain’s response to disease and explore the hypothesis that brain circuitry confers resilience or vulnerability to NeuP (Denk et al., 2014).

### The Genetic Epidemiology of NeuP

**Figure 2:**
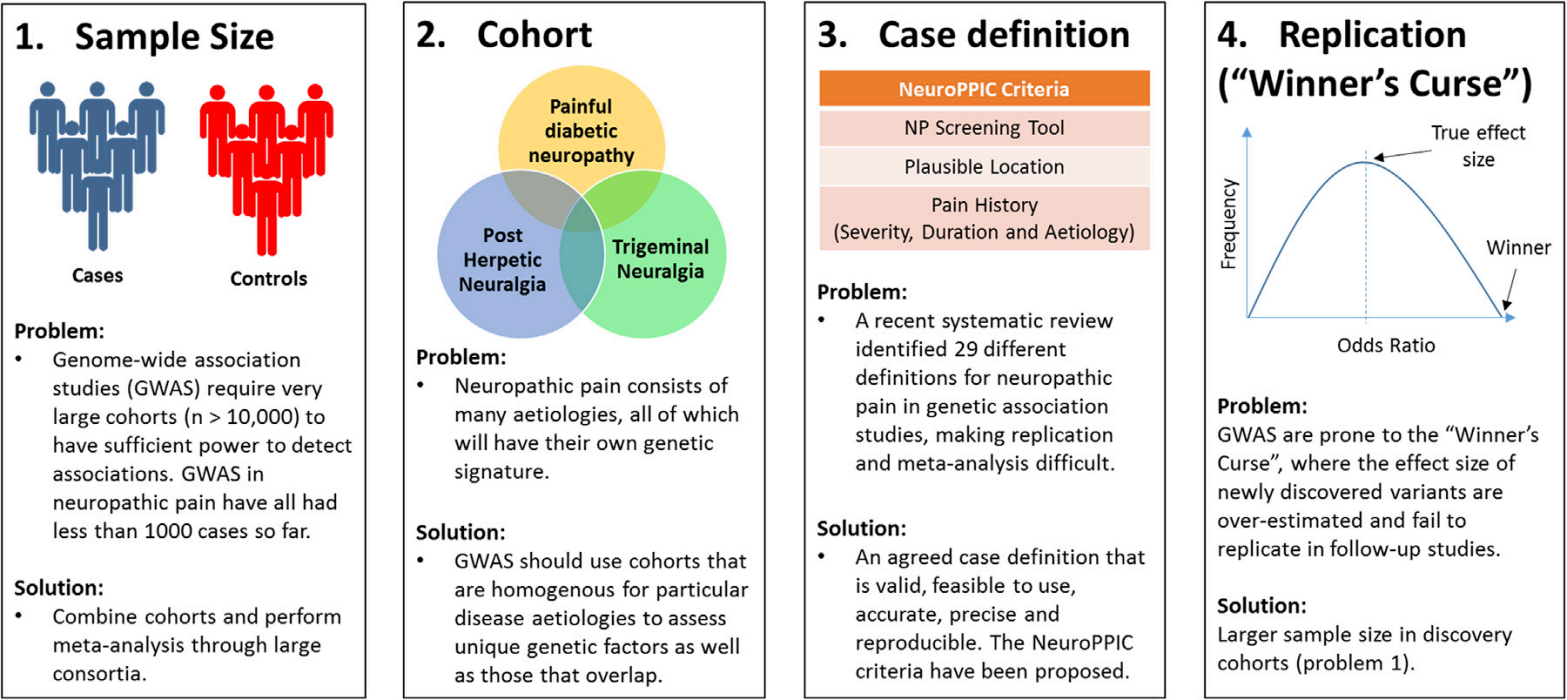
The Challenges of Conducting Genome-wide Association Studies in NeuP

Genetic epidemiology—the study of the role genetic factors play in determining health-related states or events in a population—is a useful tool to help understand NeuP as it can reveal genetic variants associated with disease risk (Manolio, 2010). Genetic epidemiology studies of NeuP have presented a number of challenges (Figure 2). The first is sample size. In order to detect genetic associations, particularly those of small effect size, a study must have sufficient statistical power. In genome-wide association studies (GWASs) of other conditions, well-powered cohorts usually exceed ten thousand participants. A recent example of this is in type 2 diabetes where one study used a cohort of 74,124 cases and 824,006 controls by combining genetic data from 32 different studies. This enabled the authors to identify 243 loci that reached significance (p < 5 × 10^−8^) and accounted for approximately 18% of the variance in risk and around a half of the estimated overall heritability (Mahajan et al., 2018). To date, genetic studies of NeuP have typically analyzed cohorts with fewer than a thousand cases, which has resulted in only suggestive associations (Hébert et al., 2017). One reason that genetic studies in NeuP lack sufficient sample sizes is the costs associated with recruiting, adequately phenotyping, and genotyping the cohort. A solution to this problem lies in cross-institution collaboration where cohorts can be combined and meta-analyses can be conducted to boost sample size and power. Combining such cohorts requires harmonization between them, which brings us onto the next challenge. A recent systematic review of genetic studies of NeuP identified 29 studies, with no two studies using the same NeuP case definition (Veluchamy et al., 2018). This has made it difficult to compare the genetic variants and their effect size estimates across studies, and to combine data to conduct meta-analyses (Evangelou and Ioannidis, 2013). A robust phenotypic definition of NeuP is needed, that all researchers adhere to, in order to accurately identify cases and controls. A consensus definition on NeuP has led to international harmony on clinical NeuP assessment, but these methods are not necessarily amenable to large populations (Finnerup et al., 2016). In a research setting, a good NeuP definition and classification into specific subgroups should be valid (identifying people with and without NeuP), feasible to use (in terms of the study time, ethics, and cost), accurate and precise (having high sensitivity and specificity), and, above all, reproducible. To address this issue, NeuP SIG have published a set of recommendations for phenotyping NeuP (van Hecke et al., 2015). The NeuP Phenotyping by International Consensus (NeuroPPIC) guidelines provide a set of entry-level criteria with which to assess participants for NeuP, including use of a validated NeuP screening tool, anatomical distribution of pain using a body chart or checklist, and pain history (including intensity, duration, underlying etiology, and demographics). However, preliminary analysis of the feasibility of NeuroPPIC suggests that it may currently be overly stringent, meaning that larger cohorts than are currently available would be required to produce a sample size that has adequate statistical power (unpublished data). This demonstrates the trade-off between feasibility and validity of phenotyping criteria. A further complication is the fact that the standard validated NeuP screening tools do not always agree. A study of the agreement of the Self-report Leeds Assessment of Neuropathic Symptoms and Signs (S-LANSS) (Bennett et al., 2005) and Douleur Neuropathique 4 Questions (DN4) (Bouhassira et al., 2005) screening tools in 45 patients with low back pain or related leg pain revealed only a moderate correlation, albeit statistically significant (Walsh et al., 2012).

**Figure 3:**
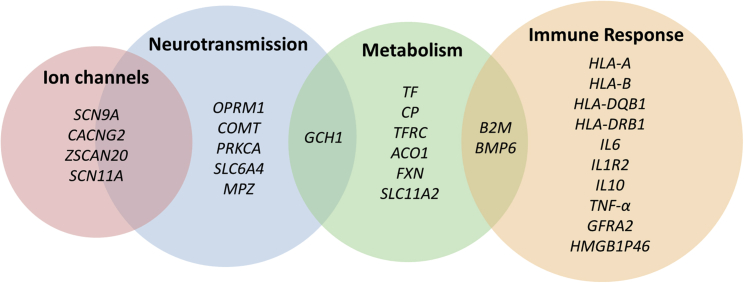
A Venn Diagram of Genes Reaching Study Specific or Suggestive Significance in Human Candidate Gene and Genome-wide Studies So Far in NeuP and the Overlap of Biological Pathways These genes have been summarized in a recent systematic review of NeuP by Veluchamy et al. (2018), where the inclusion criteria were any study analyzing genetic variants in people with NeuP compared to people without NeuP. The number of genes and our understanding of their contribution within these pathways, in the context of NeuP, is likely to change as more studies are published.

Despite these shortcomings, a number of molecular, candidate-gene, and genome-wide studies have been conducted in humans that are comprehensively summarized in a recent systematic review (Veluchamy et al., 2018). Investigations eligible for inclusion were those that examined genetic variants in people with NeuP compared to people without NeuP. These studies identified 28 genes that show study-wide association with the presence of NeuP. Together, these genes provide important clues as to the biological mechanisms involved in the onset and persistence of NeuP (Figure 3). The association of variants in *COMT*, *OPRM1*, *SCN9A*, *SLC6A4*, and *CACNG2* demonstrates the involvement of neurotransmission. Major histocompatibility complex and cytokine genes including *HLA*-*A*, *HLA*-*B*, *HLA*-*DQB1*, *HLA*-*DRB1*, *B2M*, *IL6*, *IL1R2*, *IL10*, and *TNF*-*a* represent the immune response pathway, and it is interesting to note that these genes are also associated with underlying diabetic neuropathy and post-herpetic neuralgia etiologies. Finally the identification of genes involved in iron metabolism including *ACO1*, *BMP6*, *FXN*, *TF*, *CP*, *TFRC*, *SLC11A2*, and *GCH1* illustrates the role of metabolic pathways in neuropathic sensory dysfunction. Of the genes that were identified in candidate gene-studies, variants in HLA genes, *COMT*, *OPRM1*, *TNF-a*, *IL6*, and *GCH1* were found to have an association with NeuP in more than one study (Veluchamy et al., 2018). While other genes including *SCN9A* have been associated with pain intensity in neuropathy (Reimann et al., 2010), only the *HLA* genes (*A*, *B*, and *DRB1*) have been replicated consistently as associated with the presence or absence of NeuP in the same etiology (post herpetic neuralgia) (Sato-Takeda et al., 2004, Ozawa et al., 1999, Sumiyama et al., 2008). In addition, polymorphisms in *GCH1* that were originally identified as risk factors in persistent lumbar root pain (Tegeder et al., 2006) have been associated with NeuP in a further two different etiologies (HIV-induced sensory neuropathy and persistent postsurgical pain) suggesting that the recently discovered role of *GCH1* in energy metabolism may potentially lie at a key intersection in NeuP development (Cronin et al., 2018). Furthermore, three GWASs of NeuP have been conducted to date. Two used a prescription-based phenotype for NeuP (572 and 961 cases) (Meng et al., 2015a, Meng et al., 2015b) and another used knee pain cohorts screened for NeuP using the painDETECT questionnaire (331 cases) (Warner et al., 2017). However, none of the loci identified by these studies (*GFRA2*, *ZSCAN20*-*TLR12P*, *HMGB1P46*, or *PRKCA*) reached genome-wide significance, again highlighting the importance of sample size and phenotype harmonization. Such factors are currently being addressed by the DOLORisk study (http://dolorisk.eu/) (Pascal et al., 2019), a European consortium that aims to identify risk factors for NeuP. For this study, a core group of questionnaires has been developed, based on the NeuroPPIC guidelines. This has been used to phenotype two Scottish cohorts covering 33,000 individuals. Furthermore, consenting participants of the UK Biobank cohort are currently being re-phenotyped for pain using the DN4 questionnaire (originally ∼500,000). Although the survey is still being completed, it is expected that responses will be received from around 175,000 participants. Assuming a NeuP prevalence of 7% (van Hecke et al., 2014), which is at the lower end of estimations, it is predicted that the number of people with NeuP will be over 12,000, providing the power to identify novel genetic variants.

Requiring such a stringent p value threshold (p < 5 × 10^−8^) to deal with multiple testing and protect against type I error (false association) is in tension with the greater chance of a type II error (overlooked associated genes). Pathway or gene set enrichment analysis that combines the association statistics of genes co-involved in defined signaling pathways can be one way to deal with this issue. This can be informative on the basis of aggregate data from multiple SNPs that show suggestive association but which individually do not reach the nominal threshold of 5 × 10^−8^ (Lötsch et al., 2013, Parisien et al., 2019). Another is more complex statistical approaches, and with the advent of machine learning such methods should become more common (Lötsch and Ultsch, 2018, de Los Campos et al., 2018). Large-scale genome or exome sequencing will likely become increasingly relevant in these cohorts as the price of these methods fall. As the sample sizes used in GWASs steadily increase, it is predicted that single variants will account for between one- and two-thirds of the heritability of most complex traits (Tam et al., 2019). While it is unlikely GWAS approaches will be able to explain all of NeuP heritability, it currently represents the most powerful approach available to identify genetic risk and thus targets for new therapies.

Further insights into the genetics of NeuP are being provided by the recently created Human Pain Genetics Database (HPGdb; http://humanpaingenetics.org/hpgdb) (Meloto et al., 2018). The HPGdb provides a central and up-to-date resource listing all genetic variants associated with pain and related phenotypes, including NeuP.

### How Can Genetic Epidemiology Be Used to Benefit Patients?

With more complex analysis of multiple genetic variants, attention inevitably turns to how these variants can be used to benefit patients and improve quality of life. One route being investigated is polygenic risk scores (PRSs). Polygenic risk scores quantify an individual’s overall likelihood of developing a given disorder by assessing how many known risk alleles that person has and weighting them according to their estimated effect size from GWASs. This approach has identified a significant proportion of people that have more than three times the risk of developing a disorder, for instance, defining 8% of the population at risk of coronary artery disease, 20 times more than could be identified by screening for rare monogenic disorders (Khera et al., 2018). Although there has been preliminary research into polygenic risk scores and pain, this has yet to be explored specifically in NeuP due to the lack of highly powered GWAS studies (McIntosh et al., 2016).

Currently, front-line medications used to treat NeuP, including gabapentinoids, tricyclic antidepressants, and serotonin-noradrenaline reuptake inhibitors, achieve only a 30%–50% reduction in pain severity, in 30% of patients (Finnerup et al., 2015). In order to improve clinical outcomes for existing medications and in common with patient association studies, it has been suggested that patients should be categorized according to their QST profiles (Forstenpointner et al., 2018). Furthermore, genetic variants could help explain variation in treatment response and toxicity of different drugs (pharmacogenetics). For this, variants need to be sufficiently common in the population and confer a clinically meaningful difference in treatment response. Again detecting these variants relies on large discovery cohorts, with sufficient statistical power. Because of this difficulty, very few pharmacogenetics studies have been conducted in NeuP to date (Brasch-Andersen et al., 2011, Chaudhry et al., 2017). Sensory phenotype stratification, together with classifying patients depending on genetic risk, has the potential to lead to personalized medicine to determine the best course for treatment.

### Genetic Variants in Ion Channels: From Rare Mendelian to Common NeuP Disorders

The discovery and investigation of genetic variants in ion channels is one of the best examples as to how human genetics can have a profound influence on the pain field. Hyper-excitability of the somatosensory nervous system underpins the sensory dysfunction of NeuP and is driven by the altered activity of ion channels. Mutations in ion channels have been found to underlie rare inherited painful disorders (Fertleman et al., 2006, Yang et al., 2004), and variants in the same genes have recently been associated with more common NeuP states (Faber et al., 2012a, Blesneac et al., 2018).

**Figure 4:**
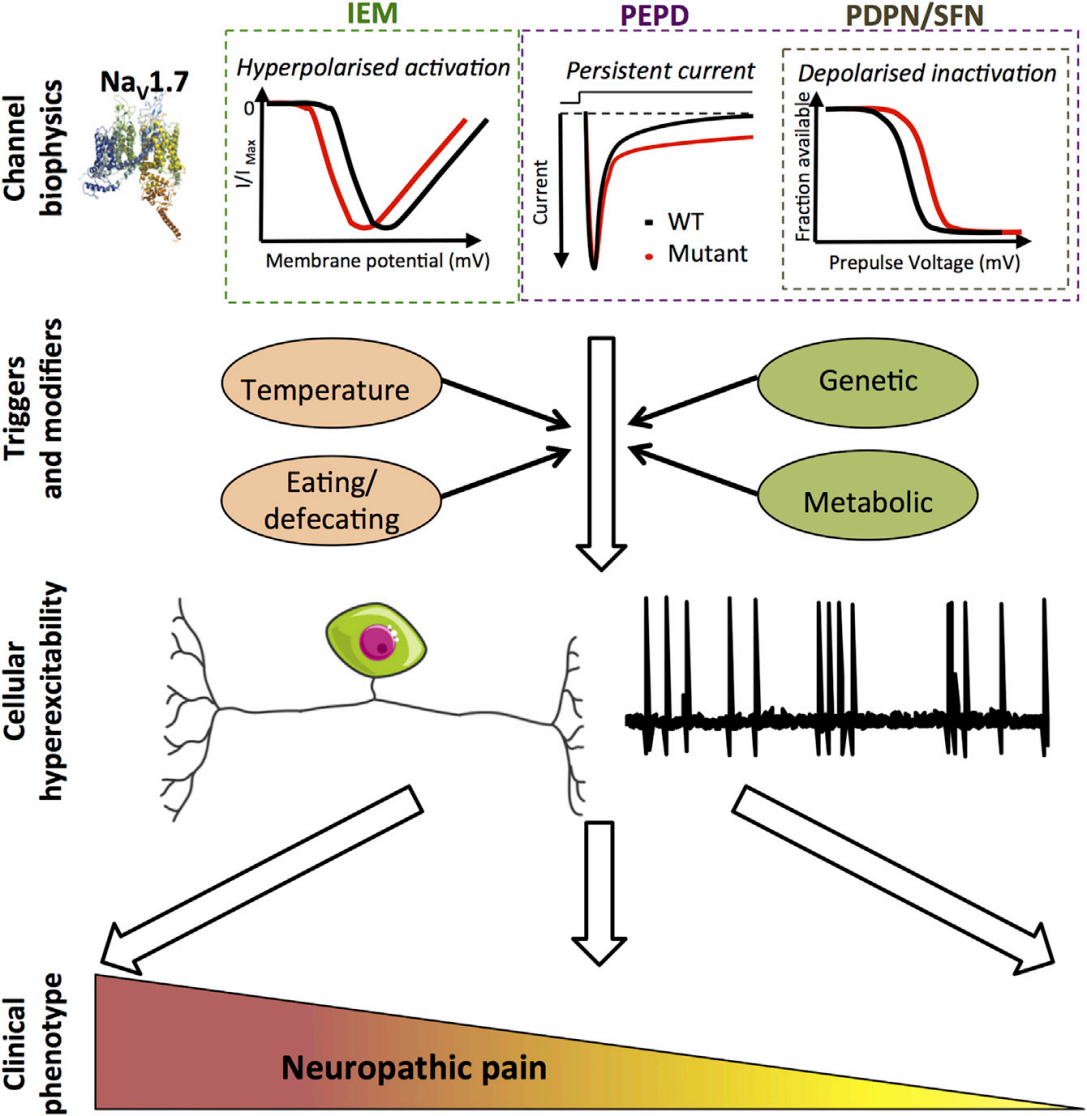
Na_V_1.7 Variants Contributing to NeuP Na_V_1.7 is the most studied ion channel with genetic association to NeuP and represents the canonical example of how variants can drive disease pathogenesis. Schematic illustrates the cardinal biophysical changes in Na_V_1.7 function associated with rare inherited (IEM and PEPD) and common NeuP states painful diabetic peripheral neuropathy (PDPN) and idiopathic small fiber neuropathy. IEM-associated variants uniquely exhibit hyperpolarized voltage of activation, whereas PEPD variants display impaired fast inactivation, which results in a persistent current in the majority of cases. Similar to PEPD, PDPN variants also demonstrate impaired fast inactivation. Small fiber neuropathy-associated variants show mixed properties, with impaired slow and fast inactivation as well as enhanced resurgent currents being described in different variants. Paroxysms of pain can be initiated by a range of triggers and are modulated by genetic and environmental factors, e.g., genetic modifiers, temperature, and metabolic state of diabetes mellitus patients (Blesneac et al., 2018, Mis et al., 2019). Convergence of these factors will determine the resultant degree of primary afferent excitability, which is important in the initiation and maintenance of NeuP. In this way, clinical outcome is dependent on the basic characteristics of the mutation interacting with modifying factors, explaining why patients with identical mutations exhibit diverse clinical phenotypes and why some mutations only result in a pain phenotype upon a secondary insult.

Most of the genetic variants that alter pain transmission have been found in three voltage-gated sodium channels: Na_V_1.7, Na_V_1.8, and Na_V_1.9. All three demonstrate highly enriched expression in pain circuits, particularly in nociceptors, and have important functions in the initiation and proper firing of action potentials (Dib-Hajj et al., 1998, Akopian et al., 1996, Bennett et al., 2019). Familial studies of rare variants with high penetrance that cause NeuP allow us to understand the contributions these channels make to primary afferent excitability. Gain-of-function mutations in *SCN9A* (the gene encoding Na_V_1.7) lead to inherited erythromelalgia (IEM; pain and erythema of the hands and feet) (Yang et al., 2004) or paroxysmal extreme pain disorder (PEPD; paroxysmal pain and erythema of the sacrum and mandible) (Fertleman et al., 2006), while loss-of-function mutations lead to pain insensitivity (Cox et al., 2006). The biophysical consequences of these mutations for channel function are summarized in Figure 4 and have been described in detail in recent reviews (Bennett et al., 2019, Dib-Hajj and Waxman, 2019).

IEM and PEPD patients carrying rare variants account for only a very small proportion of total NeuP sufferers, so how does their study advance our understanding of NeuP in general? Beyond providing insight into basic biology, familial studies have provided candidate genes to screen in common NeuP conditions. Potentially pathogenic variants in Na_V_1.7, Na_V_1.8, and Na_V_1.9 have been found in up to 17% of patients with small fiber neuropathy (Faber et al., 2012a, Faber et al., 2012b, Huang et al., 2014). In a recent study of patients with diabetic peripheral neuropathy (DPN), rare Na_V_1.7 variants were found in 9% of patients with painful, but not painless DPN (Blesneac et al., 2018), and in a separate study a single patient with painful DPN was found to carry a pathogenic mutation in Na_V_1.8 (Han et al., 2018). The biological action of these variants may be different in the two contexts. In idiopathic small fiber neuropathy (SFN), variants likely contribute directly to degeneration of intra-epidermal nerve fibers via the hyperexcitability they confer (Persson et al., 2013), whereas in DPN the presence or absence of variants appears to impact the pain experienced in the context of fibers that have already been injured by diabetes mellitus (Blesneac et al., 2018); it is also possible that such variants have an additive effect.

Given the clear role of Na_V_1.7 in inherited pain states, much effort has been invested in developing analgesic strategies targeting the channel. While still ongoing, these studies have met with mixed success. Small-molecule inhibitors have failed to deliver efficacy in phase II trials of DPN and post-herpetic neuralgia (McDonnell et al., 2018, Price et al., 2017). In contrast, one small molecule, BII074, did reach secondary endpoints and produced a degree of pain reduction for patients with trigeminal neuralgia (Zakrzewska et al., 2017). However, the specificity of this compound for Na_V_1.7 has been questioned (McDermott et al., 2019).

Developing a specific and effective Na_V_1.7 inhibitor is beset by pharmacological challenges, but, by selecting patient cohorts based on genetics, success in clinical trials may be accelerated. In the case of rare and highly penetrant mutations, this approach has already borne fruit. Studies of carbamazepine (CBZ)-responsive IEM patients described that the drug was able to reverse the hyperpolarized activation associated with the Na_V_1.7-V400M mutant, limiting the hyper-excitability of transfected dorsal root ganglion (DRG) neurons (Fischer et al., 2009). Using the V400M variant as a seed, structural modeling and thermodynamic analysis predicted CBZ sensitivity of a second IEM variant, S241T. This prediction was subsequently confirmed *in vitro*, and the strategy was validated when two patients carrying the S241T mutations reported a reduction in pain following CBZ treatment in a small double-blind trial (Geha et al., 2016). These findings have recently been extended to a Na_V_1.8 variant (Han et al., 2018). While distinct highly penetrant mutations may be rare, rational clustering of variants could provide a means to predict treatment response on an individual basis and is a realistic ambition given the increasing ease of genetic testing. In common NeuP conditions, Na_V_ inhibitors may be more effective in patients in which gain of Na_V_ channel function contributes to pathology. The anticonvulsant lacosamide is a non-selective Na_V_ channel blocker, which in early trials exhibited limited efficacy for NeuP (Finnerup et al., 2015). However, by recruiting only small fiber neuropathy patients with a Na_V_1.7 variant, a recent double-blind, placebo-controlled study demonstrated that lacosamide treatment resulted in a significant reduction in pain scores and an increase in patient well being (de Greef et al., 2019). Despite these promising results, one must be cautious not to infer clinical phenotype too strongly from gene variation. Of the 13 patients with a confirmed pathogenic Na_V_1.7 variant studied by de Greef et al., seven patients did not report a reduction in pain following lacosamide (de Greef et al., 2019). Conversely, several variants in the painful DPN cohort of Blesneac and colleagues exhibited large changes in channel biophysics, but this resulted in a pain phenotype only after a secondary insult, i.e., the metabolic complications of diabetes mellitus (Blesneac et al., 2018). These findings imply that dysfunction of an individual variant cannot alone predict clinical phenotype, nor will all patients carrying a certain variant respond to treatment equally. This lack of simple translation between genotype and phenotype is further supported by familial studies of rare variants, where pain onset and severity can vary drastically, even between family members carrying the same highly penetrant mutation (McDonnell et al., 2016), implying a significant contribution of other genetic and environmental factors to the disease phenotype (Figure 4). Defining these interactions is a key challenge and will undoubtedly require different strategies and experimental modeling of ion channel variants and indeed any genetic differences.

### Using Human Cellular Models to Study NeuP

Advances have been made in developing new cellular models capable of investigating the interaction of gene variants with phenotype, with induced pluripotent stem cell (iPSC) technology at the forefront of recent work. This approach offers the ability to investigate the functional consequence of disease-associated variants in a human neuronal cell model that retains the entire genetic background of the patient. The benefit of iPSC technology is limited by the availability of differentiation protocols to generate physiologically accurate somatic cell types, but protocols are available to generate neurons akin to nociceptors (Chambers et al., 2012). Currently, iPSC-derived nociceptor neurons are only an approximation of their endogenous counterparts and do not replicate the full panoply of sensory neuron subtypes; however, this is an ever-evolving field. Further protocols have since been published, including for the trans-differentiation of fibroblasts directly to nociceptors, which offers a larger repertoire of sensory neuron subtypes (Blanchard et al., 2015, Schrenk-Siemens et al., 2015).

In the first experiment of its kind, IEM was modeled in patient iPSC-derived nociceptors, which exhibited many features of hyper-excitability, including reduced threshold for firing and spontaneous activity (Cao et al., 2016). This study then neatly translated a novel Na_V_1.7 blocker, which reduced IEM iPSC-nociceptor excitability *in vitro* and resulted in a degree of pain reduction in donor patients. Although the study was small, there was a correlation between the patient iPSC lines that responded to the drug *in vitro*, and the patients who exhibited drug responsiveness (Cao et al., 2016). While *in vitro* phenotypes for monogenic disorders have been observed (McDermott et al., 2019, Cao et al., 2016), it remains unclear whether this approach will be sensitive enough to investigate variants with small effect sizes due to the inherent variability between iPSC lines. A recent study that compared two IEM patient lines, derived from a mother and son both carrying the Na_V_1.7 S241T mutation but who exhibited stark differences in pain severity (Mis et al., 2019), gives cause for optimism. The authors demonstrated differential excitability between the two cell lines consistent with the clinical phenotype and then, by a process of whole-exome sequencing followed by dynamic clamp studies, provided evidence of a second variant in the gene *KCNQ2* capable of modifying neuronal excitability. Such studies suggest that iPSC may enable the separation of polygenic factors underlying pain pathogenesis.

iPSC are highly amenable to CRISPR genome editing and correction of predicted pathogenic variants to generate isogenic controls is a powerful tool to causally link variants with *in vitro* phenotypes found in iPSCs (McDermott et al., 2019). This approach will be vital when investigating variants with smaller effect sizes, or variants that are highly depended on genetic background. Beyond studying genetic variants, the use of iPSC-nociceptors as screening platforms may be a substantial step forward for drug development in NeuP (Cao et al., 2016, Weir et al., 2017). Once ion channel targets are identified, drugs can be screened for their selectivity and ability to modulate neuronal excitability (Namer et al., 2019).

Cadaveric tissue and the potential to study genetic mutations directly in adult human DRG neurons represents an alternative *in vitro* approach. Such tissue is becoming more widely available, allowing for studies into cellular physiology and the contribution of key ionic currents to excitability (Zhang et al., 2017). Recent experiments have been undertaken to profile the electrophysiological responses of DRG neurons derived from NeuP patients (Li et al., 2018, North et al., 2019), opening up exciting avenues for experimentation. It may be unrealistic to consider obtaining DRG from patients with rare variants, but studies assessing DRG neurons from patients with Na_V_-related small fiber neuropathy/DPN will surely be possible in the future. It will also be possible to relate these findings in human DRG neurons to the wealth of sequencing data of mouse counterparts, which is beginning to illustrate the heterogeneity of gene expression and resultant functional properties between different sub-types of DRG neurons (Zheng et al., 2019). Defining how these individual neurons respond to nerve injury is an exciting near-term goal.

### The Use of Model Organisms to Study the Genetics of NeuP

*In silico* model systems and *in vitro* cultures of human tissues provide a novel and important way to assess the molecular interactions of any given mutation. However, as the pain experience results from integrated pathways, all the way from sensory transduction in the periphery to perception in the brain, it is critical to study this system with each level of processing intact. The development in the 1980s of transgenic and gene “knockout” mice, in combination with sequencing of the mouse genome in the early 2000s (Waterston et al., 2002) has led the advance in research into the molecular genetics of NeuP.

### Assessment of NeuP in Animal Models

The assessment of NeuP in preclinical models is associated with significant challenges given the need for indirect behavioral readouts as a surrogate of the pain experience. For practical reasons, the most commonly assessed behavioral outcomes are reflex withdrawal thresholds evoked by thermal or mechanical stimuli. Preclinical animal models of traumatic nerve injury, most often the sciatic nerve or its branches, results in evoked hypersensitivity of the animal’s hind limb (Wakisaka et al., 1991, Seltzer et al., 1990, Decosterd and Woolf, 2000, Challa, 2015). This is practically useful as most animals develop marked levels of pain-like behavior to mechanical or thermal (hot or cold) stimulation; however, there are some concerns that such models neither fully mimic traumatic nerve injury, nor reflect all aspects of nerve injury seen in the clinic. The picture of preclinical research is being enriched by using animal models beyond traumatic nerve injury to more faithfully represent NeuP inducing conditions, such as chemotherapy-induced polyneuropathies, diabetic neuropathy, multiple sclerosis, or HIV-associated peripheral neuropathy. Genetics has also been useful in developing improved models of NeuP. An example is the use of genetic mouse and rat models of diabetes which produce a slowly progressive diabetic polyneuropathy rather than the severe, acute model of streptozotocin-induced neuropathy, which is hindered by the direct neurotoxic effects of this compound (Andersson et al., 2015, Islam, 2013).

In humans, NeuP commonly presents with a complex combination of different sensory signs and symptoms. Patients with traumatic peripheral nerve injury for example show thermal hypersensitivity (increase in cold and/or heat pain sensitivity) in around 39% of cases, but only 8% of patients with polyneuropathy have this symptom (Maier et al., 2010). Mechanical hypersensitivity is seen in a third of NeuP patients with mixed etiologies; specifically blunt pressure is present in 36% of patients, pinprick sensitivity in 29%, and dynamic mechanical allodynia in 20% of those studied (Maier et al., 2010). Furthermore, evoked hypersensitivity does not correlate well with reported pain severity (Backonja and Stacey, 2004), except in certain conditions, such as post-herpetic neuralgia (Rowbotham and Fields, 1996). Thus, in contrast to mice, hypersensitivity (sensory gain) is not always present in patients and means we should not rely on any single outcome measure to inform us of the full presentation of NeuP in either species.

To address the issue of non-evoked pain in preclinical models, ethologically relevant behavioral assessment has been suggested as an indicator of ongoing pain-like behavior (Rice et al., 2008). An example in mice is burrowing behavior, which is impaired in different models of toxic neuropathies (Griffiths et al., 2018, Huang et al., 2013), although in nerve-injured animals this behavior (in addition to animal gait and grooming measures) does not correlate well with mechanical hypersensitivity (Andrews et al., 2012, Mogil et al., 2010). Conditioned place-preference/avoidance provides an external measure of internal rewarding/aversive perception (Cunningham et al., 2006) and may help to separate the sensory and affective components of the pain experience. Neuropathic animals, but not controls, prefer a chamber paired with administration of an analgesic drug, suggesting the relief of an ongoing aversive state (Tappe-Theodor et al., 2019, King et al., 2009). *In vivo* calcium imaging in awake, behaving animals was recently used to show activity in an amygdala brain circuit driven by innocuous stimuli in neuropathic animals; when this circuit was silenced, mice failed to develop conditioned place-avoidance but still displayed hypersensitive responses to evoked stimuli (Corder et al., 2019). Other cognitive behavioral assays such as thigmotaxis in an open arena, elevated plus-maze, and light-dark box preference are useful in animal studies of comorbidities of NeuP such as anxiety (Sieberg et al., 2018).

### Defining the Molecular Pathways that Create NeuP

Transcriptional profiling obtained from DRG has revealed the sheer breadth of gene regulation after neuropathic injury (Bangash et al., 2018, Chen et al., 2017) and demonstrated important contributions from non-neuronal glial and infiltrating immune cells (Cobos et al., 2018, Lopes et al., 2017b). A recent comparison of mouse and human DRG transcriptomes revealed highly conserved enrichment of transcription factors and axonal trafficking of mRNA providing strong external validity in the use of mice for drug-protein interactions (Ray et al., 2018). Conversely, regional differences in sensory neuron gene expression, such as between spinal and trigeminal sensory ganglion, could also be exploited for targeted drug discovery (Lopes et al., 2017a). Beyond mRNA, the extensive non-coding transcriptome may add a further layer of complexity to pain regulation (Baskozos et al., 2019, Zhao et al., 2013).

Sensory ganglia gene expression can also provide insight into analgesic mechanisms and potential novel analgesic therapies (Upadhyay et al., 2019). For example, variable DRG carbonic anhydrase-8 (CA8) expression appears to regulate nociception in mice through inhibiting intracellular signaling and nerve growth factor (NGF) responses (Levitt et al., 2017, Zhuang et al., 2018). Accordingly, DRG transduction of CA8 by viral gene transfer, associated with selective CA8 expression in small and medium sensory neurons, produced profound analgesia in mouse models of NeuP. Targeting sensory ganglia using gene transfer (CA8 or otherwise) may therefore represent a future therapeutic approach.

As genetic markers for subpopulations of peripheral sensory neurons and spinal cord dorsal horn neurons become better defined (Usoskin et al., 2015, Li et al., 2016, Chiu et al., 2014, Häring et al., 2018, Chamessian et al., 2018), it allows for the ever more refined targeting of specific cells involved to pain transduction. Single-cell gene profiling has been applied to tissues from neuropathy models to reveal the heterogeneity of the injury response in regeneration associated genes and cell death pathways among sensory neuron subsets (Hu et al., 2016), and expression profiling by ribosome affinity purification has revealed novel signaling pathways during chemotherapy-induced peripheral neuropathy in genetically defined sensory neurons (Megat et al., 2019). Reporter mice conditionally expressing recombinases such as Cre, Flp, Dre, and more recently VIKA (Karimova et al., 2018) can then be used to achieve highly defined expression of chemogenetic and optogenetic tools (Atasoy and Sternson, 2018, Mickle and Gereau, 2018). Used in combination with sophisticated electrophysiological preparations (Hachisuka et al., 2016), the identification of unique cell subsets in the dorsal horn, in particular, has revealed novel facets of the mechanisms of spinal gain-of-function phenomena, including “wind-up” of nociceptive inputs (Hachisuka et al., 2018) and the gate-control of innocuous mechanical stimuli evoking pain (Zhang et al., 2018).

### Genetic Homogeneity of Animal Models: A Help or a Hindrance?

The display of pain behaviors in response to any given NeuP model are generally consistent between mice of the same strain but can differ markedly *between* strains (Mogil et al., 1999). The use of genetically homogeneous inbred mice for the majority of pain research has been based on the assumption that a defined genetic background will be a less variable and more reliable model than genetically heterogeneous outbred mice (Festing, 1999). Until recently, however, little empirical evidence existed to back up this assumption (Festing, 2014). A recent comprehensive literature review revealed no difference in experimental outcomes between inbred and outbred lines. Given the same sample size, outbred mice are phenotypically as stable as inbred strains (Tuttle et al., 2018). Thus, genetically diverse outbred models should help avoid strain-specific responses and complement the study of heritable diseases across a diverse population.

In addition, inbred strains, such as C57BL/6, are commonly used to provide a homogeneous genetic background with which to test the effects of a particular gene variant on a given phenotype (Yoshiki and Moriwaki, 2006). However, the choice of genetic background can have a dramatic effect on the allele phenotype, often attributed to background-specific modifier genes or epigenetic modifications (Sittig et al., 2016). One way to control for this issue is to repeat the study in at least two different backgrounds (Linder, 2006). When genetic mutations are not stable enough to give closely matching phenotypes across strains of mice, it becomes difficult to predict their penetrance between species or in a population of patients. In common with GWAS studies, more complex algorithms to define genetic interactions and their relative importance to phenotype will undoubtedly improve our understanding of polygenic factors in animal models (de Los Campos et al., 2018, Capriotti et al., 2019).

Genetic variation also exists between mouse strains in axonal regeneration (Omura et al., 2015, Tedeschi et al., 2017). The majority of preclinical nerve injury models are designed to restrict axonal regrowth by transection or ligation of part of a peripheral nerve, leading to a permanent, partial denervation (Shields et al., 2003, Masuda et al., 2017). Recent evidence also points to a failure of damaged axons to successfully degenerate and re-innervate as a driver of persistent pain (Xie et al., 2017, Davies et al., 2019). Strain differences in the intrinsic neuronal and immune response to injury may also impact upon the development and/or resolution of the chronic pain state. Potential biases in strain-specific behavior may be overcome by use of multiple inbred strains in a single factorial design that allows a well-powered exploration of genetic heterogeneity without substantially increasing sample size (Festing et al., 2016). Alternatively, a sample of mice from a heterogeneous population such as the Diversity Outbred population may avoid the idiosyncrasies of inbred strains (Tuttle et al., 2018). The precision and allelic diversity of the outbred population greatly exceeds conventional inbred mouse strain crosses. At larger sample sizes, such populations can be used directly for precise genetic mapping of the variability in response (Recla et al., 2019).

The discovery of sex-specific effects controlling mouse NeuP development (Sorge et al., 2015, Taves et al., 2016) has highlighted the imbalance between sexes in preclinical animal models (Clayton and Collins, 2014). Sex-specific immune function has been linked to variations in pain sensitivity due to hormonal changes associated with the menstrual cycle and pregnancy (Rosen et al., 2017), or opioid analgesic efficacy (Rosen et al., 2019), and lend a mechanistic explanation to what has been suspected clinically for years (Bartley and Fillingim, 2013). Sex differences in the response to a neuropathic injury may be additionally encoded in the immune system (Lopes et al., 2017b). This raises interesting questions concerning expression profiling in chronic pain studies, since changes in gene expression could represent bona fide transcriptional responses of pain circuitry, or indicate immune cell recruitment after injury (Cobos et al., 2018, Lopes et al., 2017b, Davies et al., 2019). There are also key strain-related differences in immune function that relate to known NeuP pathways. C57BL/6 mice showed the highest vulnerability to mechanical hypersensitivity after partial nerve ligation (Isami et al., 2018); in contrast, mice of the C3H/He strain were more resistant, which correlated with more anti-inflammatory macrophages (M2) in the DRG (Isami et al., 2018). The bias of the C57BL/6 strain to Th1-type inflammatory responses (Watanabe et al., 2004) could therefore have significant bearing on neuropathies with a strong immune component.

### Reaching across the Evolutionary Divide

When considering an animal model, it is tempting to opt for fidelity, i.e., the overall likeness of a model to the human disease. Toward this end, attempts have been made to improve the relevance of preclinical models of chemotherapy-induced NeuP by climbing the evolutionary ladder from rodents to non-human primates (Hama et al., 2018). In comparison to rats, for example, macaques reportedly show a pharmacological response closer to that of patients following oxaliplatin treatment (Shidahara et al., 2016), with brain imaging revealing *de novo* activity in the somatosensory and insular cortices in response to cold stimulation only in treated animals (Nagasaka et al., 2017), similar to noxious thermal stimulation in patients undergoing oxaliplatin treatment (Boland et al., 2014).

It is the principle of discrimination—i.e., the replication of a specific phenotype in disease modeling (Russell et al., 1959)—that guides the replacement of higher-order species, such as monkeys and rodents, with lower-order species including invertebrates (Tannenbaum and Bennett, 2015). Full genome sequence assembly of *Drosophila melanogaster* (fruit fly), *Caenorhabditis elegans* (nematode worm), and *Danio rerio* (zebrafish) have allowed these organisms to play an important role in investigating conserved human disease genes and assisting drug development, despite fundamental differences in morphology and organ systems (Strynatka et al., 2018). One reason for this is that in general the function of genes, once defined, do not vary; there may be duplication and other forms of molecular evolution, but important pathways and the genes within them are by and large well conserved once their roles are established (Lander, 2011). It is also useful to consider the reasons for the first action potential in the sensory pathway: to identify and seek out the “good” (i.e., food and a healthy environment) or conversely to determine and avoid the “bad” (i.e., predation and toxic environments), the latter resulting in the creation of nociception and pain. Therefore, biology has processed noxious stimuli from the very earliest electrically excitable impulses (Smith and Lewin, 2009, Sneddon, 2018), meaning that both the pain phenotype and the genes that create it appear to be extremely well conserved across the animal phyla. Although we are a long way from attributing a perceptive quality such as “pain” to lower organisms, the use of forward genetics has led to the discovery of gene families that control nociception and more recent data hinting at the potential evolutionary conservation of neuropathic mechanisms (Khuong et al., 2019).

#### Fruit Fly

When it comes to rapid turnaround forward genetics, few organisms have made more contributions to genetic screening than the fruit fly, *Drosophila melanogaster.* The archetypal nociceptor family transient receptor potential (TRP) channels were first identified phenotypically in flies in the 1960s for their role in sensory perception of light (Cosens and Manning, 1969), and the genetic tools available in fly genetics allowed cloning of the first TRP channel in the 1980s (Montell and Rubin, 1989). The first application of fruit fly genetics to nociception was in a study by Tracey et al. (2003), who described a heat assay in fly larvae, where they exhibit a rapid rolling escape response when touched with a probe heated to 46°C.

Beyond acute nociception, the fly somatosensory system displays properties of sensitization. Initial assays focused on sensitization evoked by inflammation (Babcock et al., 2009, Babcock et al., 2011); however, models of nerve injury are now being developed (Khuong et al., 2019). Chemotherapeutic agents can sensitize larvae to mechanical and heat stimuli (Boiko et al., 2017, Brazill et al., 2018, Hamoudi et al., 2018). *Drosophila* larvae also exhibit thermal hypersensitivity following high-sugar diet or through genetic ablation of insulin signaling, and this may help in the understanding of diabetic neuropathy (Im et al., 2018).

While informative with respect to rapid sensitization, fly larval paradigms are transient by nature and cannot be used to investigate chronic sensitization. Instead, adult flies have been used for this purpose. After removal of a leg, adult fruit flies display enhanced thermal nociception escape responses and exhibit a heightened state of vigilance (Khuong et al., 2019). This disease state caused by nerve injury develops after a loss of GABAergic inhibition in the ventral nerve cord (Khuong et al., 2019), analogous to the vertebrate spinal cord. This mechanism appears to be the fly equivalent of spinal disinhibition that occurs in mice after nerve peripheral nerve injury (Mapplebeck et al., 2019). These findings establish that flies enter a NeuP-like state after injury that involves permanent central disinhibition, suggestive of chronic NeuP. A brief summary of fly paradigms is shown in Figure 1.

#### Worms and Fish

Modeling of individual characteristics and complications of peripheral neuropathies in a reductionist approach may help to define the core machinery involved in NeuP. Nematodes and zebrafish possess the neural circuitry capable of nociception, behavioral avoidance, and signal modulation (Chatigny et al., 2018) making them powerful models for high-throughput and genetic control for novel analgesic drug discovery (Ellis et al., 2018). Glucose-induced neurotoxicity in zebrafish was used to demonstrate the beneficial effect of matrix metalloproteinase (MMP) inhibition, which paralleled findings in mice (Waldron et al., 2018), suggesting the conservation of this mechanism of metabolic neuropathy. Overexpression of gain-of-function mutations of Na_V_1.7 in zebrafish sensory neurons led to decreased small fiber density and increased sensitivity to temperature changes (Eijkenboom et al., 2019), recapitulating the hallmarks of small fiber neuropathy in patients (Hoeijmakers et al., 2012).

Reverse genetics in the nematode is enhancing our understanding of hereditary peripheral neuropathies. Deposits of misfolded transthyretin (TTR) protein around sensory and autonomic nerves, characterize familial amyloid polyneuropathy with NeuP being an early symptom (Sekijima et al., 2018). Introduction of a patient TTR variant to the nematode led to a loss of noxious heat responses, likely due to the proteotoxicity of TTR oligomers (Madhivanan et al., 2018). Mutations in serine palmitoyltransferase (SPT) cause hereditary sensory and autonomic neuropathy type 1 in patients, via accumulation of neurotoxic deoxy-sphingolipids (Garofalo et al., 2011). Insertion of the equivalent SPT mutation into the nematode led to defects in neuronal polarization and vesicular trafficking (Cui et al., 2019).

### Comorbidities of NeuP

Beyond the seriously debilitating effects of chronic pain, the presence of multiple comorbidities, including anxiety, depression, and sleep disorders further complicate patient difficulties (Davis et al., 2011, Knaster et al., 2012, Radat et al., 2013). For NeuP, most work has been performed on depression (Humo et al., 2019, Doan et al., 2015, Barthas et al., 2015). Within the general population there is an incidence of depression throughout life of about 16% (Bromet et al., 2011), which compares to a rate of 50% (Bair et al., 2003) in NeuP patients, suggesting strong neurochemical commonalities. Recent data suggest that chronic pain and depression may share common genetic and cellular causes, including neuro-inflammation within the CNS (Lurie, 2018), dysregulation of neurotrophic factors and neuropeptides (Doan et al., 2015, Humo et al., 2019), as well as excessive glutamatergic, reduced GABAergic, and impaired endocannabinoid signaling (Humo et al., 2019). Microglia and astrocytes are resident non-neuronal cells of the brain and spinal cord that are important endogenous mediators of neuro-inflammation. Activation of microglia in the amygdala (Burke et al., 2013) and of astrocytes in the amygdala and periaqueductal gray (PAG) (Norman et al., 2010, Burke et al., 2013) is seen in neuropathic animals with comorbid symptoms of depression.

Anxiety in response to acute pain is responsible for hypervigilance as well as other components of the fight or flight response, and in this context it is protective. However, in NeuP these responses become chronic and can be detrimental to overall health, with widespread immune and neural dysfunction contributing to a vicious cycle of ongoing anxiety and musculoskeletal pain (Asmundson and Katz, 2009). It has been shown that anxiety levels in diabetic neuropathy patients with NeuP are significantly higher than in subjects without pain (Sieberg et al., 2018), and that NeuP presents with maximal levels of anxiety (Attal et al., 2011).

Research in animals suggests that anxiety is driven by ongoing painful input over the medium- to long-term rather than prior anxiety being a driver toward neuropathic hypersensitivity (Sieberg et al., 2018). However, anxiety can modulate pain in the short term; for example, stress-induced analgesia, which is an in-built mammalian pain suppression response that occurs during or immediately following exposure to a stressful or frightening stimulus (Butler and Finn, 2009). Therefore, measurements of anxiety levels should not be taken as a proxy for pain, unless the condition initiating the pain is adequately defined, again demonstrating the need for careful phenotyping.

Sleep disturbance is common in NeuP with estimates in chronic pain patients ranging between 50% and 80% (Cheatle et al., 2016). Interestingly, the link between sleep and pain is bi-directional, with lack of sleep amplifying pain perception (Cheatle et al., 2016, Alexandre et al., 2017). It will therefore be of interest to understand whether therapies focused on improving sleep hygiene will also improve NeuP symptoms. Sleep as a comorbidity may also be considered in relation to analgesic treatment choice for NeuP. Gabapentin/Pregabalin have been shown to improve sleep latency and depth in diabetic neuropathy (Argoff, 2007, Sabatowski et al., 2004), while conversely Duloxetine can enhance sleep fragmentation (Boyle et al., 2012).

Despite the clear associations and high prevalence of these conditions as comorbidities of NeuP, there have so far been no studies demonstrating shared genetic architecture between NeuP and depression, anxiety, or sleep disorders. However, insights can be gained from the few studies that have been conducted in chronic pain in general. A twins study, for example, found a genetic correlation of 0.56 between pain and depression and of 0.69 between pain and sleep disorders (Gasperi et al., 2017). Similarly, a family-based study found a strong genetic correlation between chronic pain and depression (r = 0.51) (McIntosh et al., 2016). Polygenic risk scores for depression were associated with chronic pain in the same cohort and in independent samples, but not vice versa (McIntosh et al., 2016). GWAS studies relating to depression, anxiety, and sleep disorders have provided some insight into the potential pleiotropic effect of the genes that have been found to be associated with NeuP in patients (Veluchamy et al., 2018) (Figure 3). For example, *HLA*-*B* is associated with feeling miserable and nervous (Nagel et al., 2018b), *HLA-DQB1* is associated with narcolepsy (Han et al., 2013) and feeling nervous or worried (Nagel et al., 2018b), and *PKRCA* is associated with neuroticism (Nagel et al., 2018a). Further work is required to uncover the genetic overlap between these comorbidities and NeuP.

### Conclusions

NeuP is diverse in its clinical phenotype, molecular mechanisms, and the types of injury that precipitate it. This diversity, in common with most chronic disease, is due to a mixture of environmental and genetic influences. In order to better treat this disparate set of pathologies, we urgently need to develop better means to stratify NeuP into accurately quantified and mechanistically relevant sub-groups (for instance, in terms of distinct sensory profiles) both in humans and in experimental animals. Only when we can accurately stratify NeuP will we be able to use whole-genome screens to define individual and shared molecular mechanisms within these populations. Such stratification will enable construction of novel therapies and better targeting of existing therapies for each chronic pain phenotype in turn. In some cases, such treatments may encompass many neuropathic etiologies; for instance, selectively reducing sodium current in peripheral nociceptors holds much promise for peripheral NeuP disorders. Clearly, the environment plays an important role in amplifying or diminishing these signaling cascades, and understanding these factors will be crucial not only for diagnosis but also for treatment. We have already made huge advances toward this goal: cross-comparing species has aided the definition of apex signaling cascades, and the use of model organisms (chiefly rodents) has been invaluable in the development of our understanding of pain processing from molecules to neural circuits and how this might be modulated with drugs or other therapies. New molecular techniques are currently turbocharging these advances: next-generation sequencing is now being applied to large clinical populations, and CRISPR technology allows rapid functional validation of identified targets in model organisms. It is our firm belief that we are poised to make a number of fundamental advances, which should aid the treatment of neuropathic and indeed other types of chronic pain. To do so, we need to continue to define the genetic architecture of NeuP, in both patients and model organisms, with the aspiration to move beyond opiates and develop new efficacious and non-addictive analgesics.

## References

[bib1] Akopian A.N., Sivilotti L., Wood J.N. (1996). A tetrodotoxin-resistant voltage-gated sodium channel expressed by sensory neurons. Nature.

[bib2] Alexandre C., Latremoliere A., Ferreira A., Miracca G., Yamamoto M., Scammell T.E., Woolf C.J. (2017). Decreased alertness due to sleep loss increases pain sensitivity in mice. Nat. Med..

[bib3] Andersson D.A., Filipović M.R., Gentry C., Eberhardt M., Vastani N., Leffler A., Reeh P., Bevan S. (2015). Streptozotocin Stimulates the Ion Channel TRPA1 Directly: Involvement of Peroxynitrite. J. Biol. Chem..

[bib4] Andrews N., Legg E., Lisak D., Issop Y., Richardson D., Harper S., Pheby T., Huang W., Burgess G., Machin I., Rice A.S. (2012). Spontaneous burrowing behaviour in the rat is reduced by peripheral nerve injury or inflammation associated pain. Eur. J. Pain.

[bib5] Argoff C.E. (2007). The coexistence of neuropathic pain, sleep, and psychiatric disorders: a novel treatment approach. Clin. J. Pain.

[bib6] Asmundson G.J., Katz J. (2009). Understanding the co-occurrence of anxiety disorders and chronic pain: state-of-the-art. Depress. Anxiety.

[bib7] Atasoy D., Sternson S.M. (2018). Chemogenetic Tools for Causal Cellular and Neuronal Biology. Physiol. Rev..

[bib8] Attal N., Lanteri-Minet M., Laurent B., Fermanian J., Bouhassira D. (2011). The specific disease burden of neuropathic pain: results of a French nationwide survey. Pain.

[bib9] Attal N., Bouhassira D., Baron R. (2018). Diagnosis and assessment of neuropathic pain through questionnaires. Lancet Neurol..

[bib10] Babcock D.T., Landry C., Galko M.J. (2009). Cytokine signaling mediates UV-induced nociceptive sensitization in Drosophila larvae. Curr. Biol..

[bib11] Babcock D.T., Shi S., Jo J., Shaw M., Gutstein H.B., Galko M.J. (2011). Hedgehog signaling regulates nociceptive sensitization. Curr. Biol..

[bib12] Backonja M.M., Stacey B. (2004). Neuropathic pain symptoms relative to overall pain rating. J. Pain.

[bib13] Bair M.J., Robinson R.L., Katon W., Kroenke K. (2003). Depression and pain comorbidity: a literature review. Arch. Intern. Med..

[bib14] Bangash M.A., Alles S.R.A., Santana-Varela S., Millet Q., Sikandar S., de Clauser L., Ter Heegde F., Habib A.M., Pereira V., Sexton J.E. (2018). Distinct transcriptional responses of mouse sensory neurons in models of human chronic pain conditions. Wellcome Open Res..

[bib15] Barthas F., Sellmeijer J., Hugel S., Waltisperger E., Barrot M., Yalcin I. (2015). The anterior cingulate cortex is a critical hub for pain-induced depression. Biol. Psychiatry.

[bib16] Bartley E.J., Fillingim R.B. (2013). Sex differences in pain: a brief review of clinical and experimental findings. Br. J. Anaesth..

[bib17] Baskozos G., Dawes J.M., Austin J.S., Antunes-Martins A., McDermott L., Clark A.J., Trendafilova T., Lees J.G., McMahon S.B., Mogil J.S. (2019). Comprehensive analysis of long noncoding RNA expression in dorsal root ganglion reveals cell-type specificity and dysregulation after nerve injury. Pain.

[bib18] Bennett M.I., Smith B.H., Torrance N., Potter J. (2005). The S-LANSS score for identifying pain of predominantly neuropathic origin: validation for use in clinical and postal research. J. Pain.

[bib19] Bennett M.I., Attal N., Backonja M.M., Baron R., Bouhassira D., Freynhagen R., Scholz J., Tölle T.R., Wittchen H.U., Jensen T.S. (2007). Using screening tools to identify neuropathic pain. Pain.

[bib20] Bennett D.L., Clark A.J., Huang J., Waxman S.G., Dib-Hajj S.D. (2019). The Role of Voltage-Gated Sodium Channels in Pain Signaling. Physiol. Rev..

[bib21] Binder A., May D., Baron R., Maier C., Tölle T.R., Treede R.D., Berthele A., Faltraco F., Flor H., Gierthmühlen J. (2011). Transient receptor potential channel polymorphisms are associated with the somatosensory function in neuropathic pain patients. PLoS ONE.

[bib22] Blanchard J.W., Eade K.T., Szűcs A., Lo Sardo V., Tsunemoto R.K., Williams D., Sanna P.P., Baldwin K.K. (2015). Selective conversion of fibroblasts into peripheral sensory neurons. Nat. Neurosci..

[bib23] Blesneac I., Themistocleous A.C., Fratter C., Conrad L.J., Ramirez J.D., Cox J.J., Tesfaye S., Shillo P.R., Rice A.S.C., Tucker S.J., Bennett D.L.H. (2018). Rare NaV1.7 variants associated with painful diabetic peripheral neuropathy. Pain.

[bib24] Boiko N., Medrano G., Montano E., Jiang N., Williams C.R., Madungwe N.B., Bopassa J.C., Kim C.C., Parrish J.Z., Hargreaves K.M. (2017). TrpA1 activation in peripheral sensory neurons underlies the ionic basis of pain hypersensitivity in response to vinca alkaloids. PLoS ONE.

[bib25] Boland E.G., Selvarajah D., Hunter M., Ezaydi Y., Tesfaye S., Ahmedzai S.H., Snowden J.A., Wilkinson I.D. (2014). Central pain processing in chronic chemotherapy-induced peripheral neuropathy: a functional magnetic resonance imaging study. PLoS ONE.

[bib26] Bouhassira D., Attal N., Alchaar H., Boureau F., Brochet B., Bruxelle J., Cunin G., Fermanian J., Ginies P., Grun-Overdyking A. (2005). Comparison of pain syndromes associated with nervous or somatic lesions and development of a new neuropathic pain diagnostic questionnaire (DN4). Pain.

[bib27] Boyle J., Eriksson M.E., Gribble L., Gouni R., Johnsen S., Coppini D.V., Kerr D. (2012). Randomized, placebo-controlled comparison of amitriptyline, duloxetine, and pregabalin in patients with chronic diabetic peripheral neuropathic pain: impact on pain, polysomnographic sleep, daytime functioning, and quality of life. Diabetes Care.

[bib28] Brasch-Andersen C., Møller M.U., Christiansen L., Thinggaard M., Otto M., Brøsen K., Sindrup S.H. (2011). A candidate gene study of serotonergic pathway genes and pain relief during treatment with escitalopram in patients with neuropathic pain shows significant association to serotonin receptor2C (HTR2C). Eur. J. Clin. Pharmacol..

[bib29] Brazill J.M., Cruz B., Zhu Y., Zhai R.G. (2018). Nmnat mitigates sensory dysfunction in a *Drosophila* model of paclitaxel-induced peripheral neuropathy. Dis. Model. Mech..

[bib30] Bromet E., Andrade L.H., Hwang I., Sampson N.A., Alonso J., de Girolamo G., de Graaf R., Demyttenaere K., Hu C., Iwata N. (2011). Cross-national epidemiology of DSM-IV major depressive episode. BMC Med..

[bib31] Burke N.N., Geoghegan E., Kerr D.M., Moriarty O., Finn D.P., Roche M. (2013). Altered neuropathic pain behaviour in a rat model of depression is associated with changes in inflammatory gene expression in the amygdala. Genes Brain Behav..

[bib32] Butler R.K., Finn D.P. (2009). Stress-induced analgesia. Prog. Neurobiol..

[bib33] Cao L., McDonnell A., Nitzsche A., Alexandrou A., Saintot P.P., Loucif A.J., Brown A.R., Young G., Mis M., Randall A. (2016). Pharmacological reversal of a pain phenotype in iPSC-derived sensory neurons and patients with inherited erythromelalgia. Sci. Transl. Med..

[bib34] Capriotti E., Ozturk K., Carter H. (2019). Integrating molecular networks with genetic variant interpretation for precision medicine. Wiley Interdiscip. Rev. Syst. Biol. Med..

[bib35] Challa S.R. (2015). Surgical animal models of neuropathic pain: Pros and Cons. Int. J. Neurosci..

[bib36] Chambers S.M., Qi Y., Mica Y., Lee G., Zhang X.J., Niu L., Bilsland J., Cao L., Stevens E., Whiting P. (2012). Combined small-molecule inhibition accelerates developmental timing and converts human pluripotent stem cells into nociceptors. Nat. Biotechnol..

[bib37] Chamessian A., Young M., Qadri Y., Berta T., Ji R.R., Van de Ven T. (2018). Transcriptional Profiling of Somatostatin Interneurons in the Spinal Dorsal Horn. Sci. Rep..

[bib38] Chatigny F., Creighton C.M., Stevens E.D. (2018). Updated Review of Fish Analgesia. J. Am. Assoc. Lab. Anim. Sci..

[bib39] Chaudhry M., Alessandrini M., Rademan J., Dodgen T.M., Steffens F.E., van Zyl D.G., Gaedigk A., Pepper M.S. (2017). Impact of CYP2D6 genotype on amitriptyline efficacy for the treatment of diabetic peripheral neuropathy: a pilot study. Pharmacogenomics.

[bib40] Cheatle M.D., Foster S., Pinkett A., Lesneski M., Qu D., Dhingra L. (2016). Assessing and Managing Sleep Disturbance in Patients with Chronic Pain. Sleep Med. Clin..

[bib41] Chen C.J., Liu D.Z., Yao W.F., Gu Y., Huang F., Hei Z.Q., Li X. (2017). Identification of key genes and pathways associated with neuropathic pain in uninjured dorsal root ganglion by using bioinformatic analysis. J. Pain Res..

[bib42] Chiu I.M., Barrett L.B., Williams E.K., Strochlic D.E., Lee S., Weyer A.D., Lou S., Bryman G.S., Roberson D.P., Ghasemlou N. (2014). Transcriptional profiling at whole population and single cell levels reveals somatosensory neuron molecular diversity. eLife.

[bib43] Clayton J.A., Collins F.S. (2014). Policy: NIH to balance sex in cell and animal studies. Nature.

[bib44] Cobos E.J., Nickerson C.A., Gao F., Chandran V., Bravo-Caparrós I., González-Cano R., Riva P., Andrews N.A., Latremoliere A., Seehus C.R. (2018). Mechanistic Differences in Neuropathic Pain Modalities Revealed by Correlating Behavior with Global Expression Profiling. Cell Rep..

[bib45] Corder G., Ahanonu B., Grewe B.F., Wang D., Schnitzer M.J., Scherrer G. (2019). An amygdalar neural ensemble that encodes the unpleasantness of pain. Science.

[bib46] Cosens D.J., Manning A. (1969). Abnormal electroretinogram from a Drosophila mutant. Nature.

[bib47] Cox J.J., Reimann F., Nicholas A.K., Thornton G., Roberts E., Springell K., Karbani G., Jafri H., Mannan J., Raashid Y. (2006). An SCN9A channelopathy causes congenital inability to experience pain. Nature.

[bib48] Cronin S.J.F., Seehus C., Weidinger A., Talbot S., Reissig S., Seifert M., Pierson Y., McNeill E., Longhi M.S., Turnes B.L. (2018). The metabolite BH4 controls T cell proliferation in autoimmunity and cancer. Nature.

[bib49] Cui M., Ying R., Jiang X., Li G., Zhang X., Zheng J., Tam K.Y., Liang B., Shi A., Göbel V., Zhang H. (2019). A Model of Hereditary Sensory and Autonomic Neuropathy Type 1 Reveals a Role of Glycosphingolipids in Neuronal Polarity. J. Neurosci..

[bib50] Cunningham C.L., Gremel C.M., Groblewski P.A. (2006). Drug-induced conditioned place preference and aversion in mice. Nat. Protoc..

[bib51] Davies A.J., Kim H.W., Gonzalez-Cano R., Choi J., Back S.K., Roh S.E., Johnson E., Gabriac M., Kim M.S., Lee J. (2019). Natural Killer Cells Degenerate Intact Sensory Afferents following Nerve Injury. Cell.

[bib52] Davis J.A., Robinson R.L., Le T.K., Xie J. (2011). Incidence and impact of pain conditions and comorbid illnesses. J. Pain Res..

[bib53] de Greef B.T.A., Hoeijmakers J.G.J., Geerts M., Oakes M., Church T.J.E., Waxman S.G., Dib-Hajj S.D., Faber C.G., Merkies I.S.J. (2019). Lacosamide in patients with Nav1.7 mutations-related small fibre neuropathy: a randomized controlled trial. Brain.

[bib54] de Los Campos G., Vazquez A.I., Hsu S., Lello L. (2018). Complex-Trait Prediction in the Era of Big Data. Trends Genet..

[bib55] Decosterd I., Woolf C.J. (2000). Spared nerve injury: an animal model of persistent peripheral neuropathic pain. Pain.

[bib56] Denk F., McMahon S.B., Tracey I. (2014). Pain vulnerability: a neurobiological perspective. Nat. Neurosci..

[bib57] Dib-Hajj S.D., Waxman S.G. (2019). Sodium Channels in Human Pain Disorders: Genetics and Pharmacogenomics. Annu. Rev. Neurosci..

[bib58] Dib-Hajj S.D., Tyrrell L., Black J.A., Waxman S.G. (1998). NaN, a novel voltage-gated Na channel, is expressed preferentially in peripheral sensory neurons and down-regulated after axotomy. Proc. Natl. Acad. Sci. USA.

[bib59] Doan L., Manders T., Wang J. (2015). Neuroplasticity underlying the comorbidity of pain and depression. Neural Plast..

[bib60] Eijkenboom I., Sopacua M., Otten A.B.C., Gerrits M.M., Hoeijmakers J.G.J., Waxman S.G., Lombardi R., Lauria G., Merkies I.S.J., Smeets H.J.M., PROPANE Study Group (2019). Expression of pathogenic SCN9A mutations in the zebrafish: A model to study small-fiber neuropathy. Exp. Neurol..

[bib61] Elliott L.T., Sharp K., Alfaro-Almagro F., Shi S., Miller K.L., Douaud G., Marchini J., Smith S.M. (2018). Genome-wide association studies of brain imaging phenotypes in UK Biobank. Nature.

[bib62] Ellis L.D., Berrue F., Morash M., Achenbach J.C., Hill J., McDougall J.J. (2018). Comparison of cannabinoids with known analgesics using a novel high throughput zebrafish larval model of nociception. Behav. Brain Res..

[bib63] Evangelou E., Ioannidis J.P.A. (2013). Meta-analysis methods for genome-wide association studies and beyond. Nat. Rev. Genet..

[bib64] Faber C.G., Hoeijmakers J.G., Ahn H.S., Cheng X., Han C., Choi J.S., Estacion M., Lauria G., Vanhoutte E.K., Gerrits M.M. (2012). Gain of function Naν1.7 mutations in idiopathic small fiber neuropathy. Ann. Neurol..

[bib65] Faber C.G., Lauria G., Merkies I.S., Cheng X., Han C., Ahn H.S., Persson A.K., Hoeijmakers J.G., Gerrits M.M., Pierro T. (2012). Gain-of-function Nav1.8 mutations in painful neuropathy. Proc. Natl. Acad. Sci. USA.

[bib66] Feldman E.L., Nave K.A., Jensen T.S., Bennett D.L.H. (2017). New Horizons in Diabetic Neuropathy: Mechanisms, Bioenergetics, and Pain. Neuron.

[bib67] Fertleman C.R., Baker M.D., Parker K.A., Moffatt S., Elmslie F.V., Abrahamsen B., Ostman J., Klugbauer N., Wood J.N., Gardiner R.M., Rees M. (2006). SCN9A mutations in paroxysmal extreme pain disorder: allelic variants underlie distinct channel defects and phenotypes. Neuron.

[bib68] Festing M.F. (1999). Warning: the use of heterogeneous mice may seriously damage your research. Neurobiol. Aging.

[bib69] Festing M.F. (2014). Evidence should trump intuition by preferring inbred strains to outbred stocks in preclinical research. ILAR J..

[bib70] Festing M.F.W., Overend P., Borja M.C., Berdoy M. (2016). The Design of Animal Experiments: Reducing the Use of Animals in Research through Better Experimental Design.

[bib71] Finnerup N.B., Attal N., Haroutounian S., McNicol E., Baron R., Dworkin R.H., Gilron I., Haanpää M., Hansson P., Jensen T.S. (2015). Pharmacotherapy for neuropathic pain in adults: a systematic review and meta-analysis. Lancet Neurol..

[bib72] Finnerup N.B., Haroutounian S., Kamerman P., Baron R., Bennett D.L.H., Bouhassira D., Cruccu G., Freeman R., Hansson P., Nurmikko T. (2016). Neuropathic pain: an updated grading system for research and clinical practice. Pain.

[bib73] Finnerup N.B., Haroutounian S., Baron R., Dworkin R.H., Gilron I., Haanpaa M., Jensen T.S., Kamerman P.R., McNicol E., Moore A. (2018). Neuropathic pain clinical trials: factors associated with decreases in estimated drug efficacy. Pain.

[bib74] Fischer T.Z., Gilmore E.S., Estacion M., Eastman E., Taylor S., Melanson M., Dib-Hajj S.D., Waxman S.G. (2009). A novel Nav1.7 mutation producing carbamazepine-responsive erythromelalgia. Ann. Neurol..

[bib75] Forstenpointner J., Otto J., Baron R. (2018). Individualized neuropathic pain therapy based on phenotyping: are we there yet?. Pain.

[bib76] Garofalo K., Penno A., Schmidt B.P., Lee H.J., Frosch M.P., von Eckardstein A., Brown R.H., Hornemann T., Eichler F.S. (2011). Oral L-serine supplementation reduces production of neurotoxic deoxysphingolipids in mice and humans with hereditary sensory autonomic neuropathy type 1. J. Clin. Invest..

[bib77] Gasperi M., Herbert M., Schur E., Buchwald D., Afari N. (2017). Genetic and Environmental Influences on Sleep, Pain, and Depression Symptoms in a Community Sample of Twins. Psychosom. Med..

[bib78] GBD 2017 Disease and Injury Incidence and Prevalence Collaborators (2018). Global, regional, and national incidence, prevalence, and years lived with disability for 354 diseases and injuries for 195 countries and territories, 1990–2017: a systematic analysis for the Global Burden of Disease Study 2017. Lancet.

[bib79] Geha P., Yang Y., Estacion M., Schulman B.R., Tokuno H., Apkarian A.V., Dib-Hajj S.D., Waxman S.G. (2016). Pharmacotherapy for Pain in a Family With Inherited Erythromelalgia Guided by Genomic Analysis and Functional Profiling. JAMA Neurol..

[bib80] Griffiths L.A., Duggett N.A., Pitcher A.L., Flatters S.J.L. (2018). Evoked and Ongoing Pain-Like Behaviours in a Rat Model of Paclitaxel-Induced Peripheral Neuropathy. Pain Res. Manag..

[bib81] Hachisuka J., Baumbauer K.M., Omori Y., Snyder L.M., Koerber H.R., Ross S.E. (2016). Semi-intact ex vivo approach to investigate spinal somatosensory circuits. eLife.

[bib82] Hachisuka J., Omori Y., Chiang M.C., Gold M.S., Koerber H.R., Ross S.E. (2018). Wind-up in lamina I spinoparabrachial neurons: a role for reverberatory circuits. Pain.

[bib83] Hama A., Natsume T., Ogawa S., Higo N., Hayashi I., Takamatsu H. (2018). Gaps in Understanding Mechanism and Lack of Treatments: Potential Use of a Nonhuman Primate Model of Oxaliplatin-Induced Neuropathic Pain. Pain Res. Manag..

[bib84] Hamoudi Z., Khuong T.M., Cole T., Neely G.G. (2018). A fruit fly model for studying paclitaxel-induced peripheral neuropathy and hyperalgesia. F1000Res..

[bib85] Han F., Faraco J., Dong X.S., Ollila H.M., Lin L., Li J., An P., Wang S., Jiang K.W., Gao Z.C. (2013). Genome wide analysis of narcolepsy in China implicates novel immune loci and reveals changes in association prior to versus after the 2009 H1N1 influenza pandemic. PLoS Genet..

[bib86] Han C., Themistocleous A.C., Estacion M., Dib-Hajj F.B., Blesneac I., Macala L., Fratter C., Bennett D.L., Waxman S.G., Dib-Hajj S.D. (2018). The Novel Activity of Carbamazepine as an Activation Modulator Extends from Na_V_1.7 Mutations to the Na_V_1.8-S242T Mutant Channel from a Patient with Painful Diabetic Neuropathy. Mol. Pharmacol..

[bib87] Häring M., Zeisel A., Hochgerner H., Rinwa P., Jakobsson J.E.T., Lönnerberg P., La Manno G., Sharma N., Borgius L., Kiehn O. (2018). Neuronal atlas of the dorsal horn defines its architecture and links sensory input to transcriptional cell types. Nat. Neurosci..

[bib88] Hébert H.L., Veluchamy A., Torrance N., Smith B.H. (2017). Risk factors for neuropathic pain in diabetes mellitus. Pain.

[bib89] Heiman R.G., Atkinson R.C., Andruss B.F., Bolduc C., Kovalick G.E., Beckingham K. (1996). Spontaneous avoidance behavior in Drosophila null for calmodulin expression. Proc. Natl. Acad. Sci. USA.

[bib90] Hoeijmakers J.G., Han C., Merkies I.S., Macala L.J., Lauria G., Gerrits M.M., Dib-Hajj S.D., Faber C.G., Waxman S.G. (2012). Small nerve fibres, small hands and small feet: a new syndrome of pain, dysautonomia and acromesomelia in a kindred with a novel NaV1.7 mutation. Brain.

[bib91] Hu G., Huang K., Hu Y., Du G., Xue Z., Zhu X., Fan G. (2016). Single-cell RNA-seq reveals distinct injury responses in different types of DRG sensory neurons. Sci. Rep..

[bib92] Huang W., Calvo M., Karu K., Olausen H.R., Bathgate G., Okuse K., Bennett D.L., Rice A.S. (2013). A clinically relevant rodent model of the HIV antiretroviral drug stavudine induced painful peripheral neuropathy. Pain.

[bib93] Huang J., Han C., Estacion M., Vasylyev D., Hoeijmakers J.G., Gerrits M.M., Tyrrell L., Lauria G., Faber C.G., Dib-Hajj S.D., PROPANE Study Group (2014). Gain-of-function mutations in sodium channel Na(v)1.9 in painful neuropathy. Brain.

[bib94] Humo M., Lu H., Yalcin I. (2019). The molecular neurobiology of chronic pain-induced depression. Cell Tissue Res..

[bib95] Im S.H., Patel A.A., Cox D.N., Galko M.J. (2018). *Drosophila* Insulin receptor regulates the persistence of injury-induced nociceptive sensitization. Dis. Model. Mech..

[bib96] Isami K., Imai S., Sukeishi A., Nagayasu K., Shirakawa H., Nakagawa T., Kaneko S. (2018). The impact of mouse strain-specific spatial and temporal immune responses on the progression of neuropathic pain. Brain Behav. Immun..

[bib97] Islam M.S. (2013). Animal models of diabetic neuropathy: progress since 1960s. J. Diabetes Res..

[bib98] Jensen T.S., Baron R., Haanpää M., Kalso E., Loeser J.D., Rice A.S.C., Treede R.-D. (2011). A new definition of neuropathic pain. Pain.

[bib99] Jones M.R., Viswanath O., Peck J., Kaye A.D., Gill J.S., Simopoulos T.T. (2018). A Brief History of the Opioid Epidemic and Strategies for Pain Medicine. Pain Ther..

[bib100] Karimova M., Baker O., Camgoz A., Naumann R., Buchholz F., Anastassiadis K. (2018). A single reporter mouse line for Vika, Flp, Dre, and Cre-recombination. Sci. Rep..

[bib101] Kennedy D.L., Kemp H.I., Ridout D., Yarnitsky D., Rice A.S. (2016). Reliability of conditioned pain modulation: a systematic review. Pain.

[bib102] Khera A.V., Chaffin M., Aragam K.G., Haas M.E., Roselli C., Choi S.H., Natarajan P., Lander E.S., Lubitz S.A., Ellinor P.T., Kathiresan S. (2018). Genome-wide polygenic scores for common diseases identify individuals with risk equivalent to monogenic mutations. Nat. Genet..

[bib103] Khuong T.M., Wang Q.P., Manion J., Oyston L.J., Lau M.T., Towler H., Lin Y.Q., Neely G.G. (2019). Nerve injury drives a heightened state of vigilance and neuropathic sensitization in Drosophila. Sci. Adv..

[bib104] King T., Vera-Portocarrero L., Gutierrez T., Vanderah T.W., Dussor G., Lai J., Fields H.L., Porreca F. (2009). Unmasking the tonic-aversive state in neuropathic pain. Nat. Neurosci..

[bib105] Knaster P., Estlander A.M., Karlsson H., Kaprio J., Kalso E. (2012). Temperament traits and chronic pain: the association of harm avoidance and pain-related anxiety. PLoS ONE.

[bib106] Lander E.S. (2011). Initial impact of the sequencing of the human genome. Nature.

[bib107] Levitt R.C., Zhuang G.Y., Kang Y., Erasso D.M., Upadhyay U., Ozdemir M., Fu E.S., Sarantopoulos K.D., Smith S.B., Maixner W. (2017). Car8 dorsal root ganglion expression and genetic regulation of analgesic responses are associated with a cis-eQTL in mice. Mamm. Genome.

[bib108] Li C.L., Li K.C., Wu D., Chen Y., Luo H., Zhao J.R., Wang S.S., Sun M.M., Lu Y.J., Zhong Y.Q. (2016). Somatosensory neuron types identified by high-coverage single-cell RNA-sequencing and functional heterogeneity. Cell Res..

[bib109] Li Y., North R.Y., Rhines L.D., Tatsui C.E., Rao G., Edwards D.D., Cassidy R.M., Harrison D.S., Johansson C.A., Zhang H., Dougherty P.M. (2018). DRG Voltage-Gated Sodium Channel 1.7 Is Upregulated in Paclitaxel-Induced Neuropathy in Rats and in Humans with Neuropathic Pain. J. Neurosci..

[bib110] Linder C.C. (2006). Genetic variables that influence phenotype. ILAR J..

[bib111] Lopes D.M., Denk F., McMahon S.B. (2017). The Molecular Fingerprint of Dorsal Root and Trigeminal Ganglion Neurons. Front. Mol. Neurosci..

[bib112] Lopes D.M., Malek N., Edye M., Jager S.B., McMurray S., McMahon S.B., Denk F. (2017). Sex differences in peripheral not central immune responses to pain-inducing injury. Sci. Rep..

[bib113] Lötsch J., Ultsch A. (2018). Machine learning in pain research. Pain.

[bib114] Lötsch J., Doehring A., Mogil J.S., Arndt T., Geisslinger G., Ultsch A. (2013). Functional genomics of pain in analgesic drug development and therapy. Pharmacol. Ther..

[bib115] Lurie D.I. (2018). An Integrative Approach to Neuroinflammation in Psychiatric disorders and Neuropathic Pain. J. Exp. Neurosci..

[bib116] Madhivanan K., Greiner E.R., Alves-Ferreira M., Soriano-Castell D., Rouzbeh N., Aguirre C.A., Paulsson J.F., Chapman J., Jiang X., Ooi F.K. (2018). Cellular clearance of circulating transthyretin decreases cell-nonautonomous proteotoxicity in *Caenorhabditis elegans*. Proc. Natl. Acad. Sci. USA.

[bib117] Mahajan A., Taliun D., Thurner M., Robertson N.R., Torres J.M., Rayner N.W., Payne A.J., Steinthorsdottir V., Scott R.A., Grarup N. (2018). Fine-mapping type 2 diabetes loci to single-variant resolution using high-density imputation and islet-specific epigenome maps. Nat. Genet..

[bib118] Maier C., Baron R., Tölle T.R., Binder A., Birbaumer N., Birklein F., Gierthmühlen J., Flor H., Geber C., Huge V. (2010). Quantitative sensory testing in the German Research Network on Neuropathic Pain (DFNS): somatosensory abnormalities in 1236 patients with different neuropathic pain syndromes. Pain.

[bib119] Manolio T.A. (2010). Genomewide association studies and assessment of the risk of disease. N. Engl. J. Med..

[bib120] Mapplebeck J.C.S., Lorenzo L.E., Lee K.Y., Gauthier C., Muley M.M., De Koninck Y., Prescott S.A., Salter M.W. (2019). Chloride Dysregulation through Downregulation of KCC2 Mediates Neuropathic Pain in Both Sexes. Cell Rep..

[bib121] Masuda T., Kohro Y., Inoue K., Tsuda M. (2017).

[bib122] McDermott L.A., Weir G.A., Themistocleous A.C., Segerdahl A.R., Blesneac I., Baskozos G., Clark A.J., Millar V., Peck L.J., Ebner D. (2019). Defining the Functional Role of NaV1.7 in Human Nociception. Neuron.

[bib123] McDonnell A., Schulman B., Ali Z., Dib-Hajj S.D., Brock F., Cobain S., Mainka T., Vollert J., Tarabar S., Waxman S.G. (2016). Inherited erythromelalgia due to mutations in SCN9A: natural history, clinical phenotype and somatosensory profile. Brain.

[bib124] McDonnell A., Collins S., Ali Z., Iavarone L., Surujbally R., Kirby S., Butt R.P. (2018). Efficacy of the Nav1.7 blocker PF-05089771 in a randomised, placebo-controlled, double-blind clinical study in subjects with painful diabetic peripheral neuropathy. Pain.

[bib125] McIntosh A.M., Hall L.S., Zeng Y., Adams M.J., Gibson J., Wigmore E., Hagenaars S.P., Davies G., Fernandez-Pujals A.M., Campbell A.I. (2016). Genetic and Environmental Risk for Chronic Pain and the Contribution of Risk Variants for Major Depressive Disorder: A Family-Based Mixed-Model Analysis. PLoS Med..

[bib126] Megat S., Ray P.R., Moy J.K., Lou T.F., Barragán-Iglesias P., Li Y., Pradhan G., Wanghzou A., Ahmad A., Burton M.D. (2019). Nociceptor Translational Profiling Reveals the Ragulator-Rag GTPase Complex as a Critical Generator of Neuropathic Pain. J. Neurosci..

[bib127] Meloto C.B., Benavides R., Lichtenwalter R.N., Wen X., Tugarinov N., Zorina-Lichtenwalter K., Chabot-Doré A.-J., Piltonen M.H., Cattaneo S., Verma V. (2018). Human pain genetics database: a resource dedicated to human pain genetics research. Pain.

[bib128] Meng W., Deshmukh H.A., Donnelly L.A., Torrance N., Colhoun H.M., Palmer C.N., Smith B.H., Wellcome Trust Case Control Consortium 2 (WTCCC2), Surrogate markers for Micro- and Macro-vascular hard endpoints for Innovative diabetes Tools (SUMMIT) study group (2015). A genome-wide association study provides evidence of sex-specific involvement of chr1p35.1 (ZSCAN20-TLR12P) and chr8p23.1 (HMGB1P46) with diabetic neuropathic pain. EBioMedicine.

[bib129] Meng W., Deshmukh H.A., van Zuydam N.R., Liu Y., Donnelly L.A., Zhou K., Morris A.D., Colhoun H.M., Palmer C.N., Smith B.H., Wellcome Trust Case Control Consortium 2 (WTCCC2), Surrogate Markers for Micro- and Macro-Vascular Hard Endpoints for Innovative Diabetes Tools (SUMMIT) Study Group (2015). A genome-wide association study suggests an association of Chr8p21.3 (GFRA2) with diabetic neuropathic pain. Eur. J. Pain.

[bib130] Mickle A.D., Gereau R.W. (2018). A bright future? Optogenetics in the periphery for pain research and therapy. Pain.

[bib131] Mis M.A., Yang Y., Tanaka B.S., Gomis-Perez C., Liu S., Dib-Hajj F., Adi T., Garcia-Milian R., Schulman B.R., Dib-Hajj S.D., Waxman S.G. (2019). Resilience to Pain: A Peripheral Component Identified Using Induced Pluripotent Stem Cells and Dynamic Clamp. J. Neurosci..

[bib132] Mogil J.S., Wilson S.G., Bon K., Lee S.E., Chung K., Raber P., Pieper J.O., Hain H.S., Belknap J.K., Hubert L. (1999). Heritability of nociception I: responses of 11 inbred mouse strains on 12 measures of nociception. Pain.

[bib133] Mogil J.S., Graham A.C., Ritchie J., Hughes S.F., Austin J.S., Schorscher-Petcu A., Langford D.J., Bennett G.J. (2010). Hypolocomotion, asymmetrically directed behaviors (licking, lifting, flinching, and shaking) and dynamic weight bearing (gait) changes are not measures of neuropathic pain in mice. Mol. Pain.

[bib134] Montell C., Rubin G.M. (1989). Molecular characterization of the Drosophila trp locus: a putative integral membrane protein required for phototransduction. Neuron.

[bib135] Nagasaka K., Yamanaka K., Ogawa S., Takamatsu H., Higo N. (2017). Brain activity changes in a macaque model of oxaliplatin-induced neuropathic cold hypersensitivity. Sci. Rep..

[bib136] Nagel M., Jansen P.R., Stringer S., Watanabe K., de Leeuw C.A., Bryois J., Savage J.E., Hammerschlag A.R., Skene N.G., Muñoz-Manchado A.B., 23andMe Research Team (2018). Meta-analysis of genome-wide association studies for neuroticism in 449,484 individuals identifies novel genetic loci and pathways. Nat. Genet..

[bib137] Nagel M., Watanabe K., Stringer S., Posthuma D., van der Sluis S. (2018). Item-level analyses reveal genetic heterogeneity in neuroticism. Nat. Commun..

[bib138] Namer B., Schmidt D., Eberhardt E., Maroni M., Dorfmeister E., Kleggetveit I.P., Kaluza L., Meents J., Gerlach A., Lin Z. (2019). Pain relief in a neuropathy patient by lacosamide: Proof of principle of clinical translation from patient-specific iPS cell-derived nociceptors. EBioMedicine.

[bib139] Norman G.J., Karelina K., Zhang N., Walton J.C., Morris J.S., Devries A.C. (2010). Stress and IL-1beta contribute to the development of depressive-like behavior following peripheral nerve injury. Mol. Psychiatry.

[bib140] North R.Y., Li Y., Ray P., Rhines L.D., Tatsui C.E., Rao G., Johansson C.A., Zhang H., Kim Y.H., Zhang B. (2019). Electrophysiological and transcriptomic correlates of neuropathic pain in human dorsal root ganglion neurons. Brain.

[bib141] Omura T., Omura K., Tedeschi A., Riva P., Painter M.W., Rojas L., Martin J., Lisi V., Huebner E.A., Latremoliere A. (2015). Robust Axonal Regeneration Occurs in the Injured CAST/Ei Mouse CNS. Neuron.

[bib142] Ozawa A., Sasao Y., Iwashita K., Miyahara M., Sugai J., Iizuka M., Kawakubo Y., Ohkido M., Naruse T., Anzai T. (1999). HLA-A33 and -B44 and susceptibility to postherpetic neuralgia (PHN). Tissue Antigens.

[bib143] Parisien M., Samoshkin A., Tansley S.N., Piltonen M.H., Martin L.J., El-Hachem N., Dagostino C., Allegri M., Mogil J.S., Khoutorsky A., Diatchenko L. (2019). Genetic pathway analysis reveals a major role for extracellular matrix organization in inflammatory and neuropathic pain. Pain.

[bib144] Pascal M.M.V., Themistocleous A.C., Baron R., Binder A., Bouhassira D., Crombez G., Finnerup N.B., Gierthmühlen J., Granovsky Y., Groop L. (2019). DOLORisk: study protocol for a multi-centre observational study to understand the risk factors and determinants of neuropathic pain. Wellcome Open Res..

[bib145] Percie du Sert N., Rice A.S. (2014). Improving the translation of analgesic drugs to the clinic: animal models of neuropathic pain. Br. J. Pharmacol..

[bib146] Persson A.K., Liu S., Faber C.G., Merkies I.S., Black J.A., Waxman S.G. (2013). Neuropathy-associated Nav1.7 variant I228M impairs integrity of dorsal root ganglion neuron axons. Ann. Neurol..

[bib147] Price N., Namdari R., Neville J., Proctor K.J., Kaber S., Vest J., Fetell M., Malamut R., Sherrington R.P., Pimstone S.N., Goldberg Y.P. (2017). Safety and Efficacy of a Topical Sodium Channel Inhibitor (TV-45070) in Patients With Postherpetic Neuralgia (PHN): A Randomized, Controlled, Proof-of-Concept, Crossover Study, With a Subgroup Analysis of the Nav1.7 R1150W Genotype. Clin. J. Pain.

[bib148] Radat F., Margot-Duclot A., Attal N. (2013). Psychiatric co-morbidities in patients with chronic peripheral neuropathic pain: a multicentre cohort study. Eur. J. Pain.

[bib149] Ray P., Torck A., Quigley L., Wangzhou A., Neiman M., Rao C., Lam T., Kim J.Y., Kim T.H., Zhang M.Q. (2018). Comparative transcriptome profiling of the human and mouse dorsal root ganglia: an RNA-seq-based resource for pain and sensory neuroscience research. Pain.

[bib150] Recla J.M., Bubier J.A., Gatti D.M., Ryan J.L., Long K.H., Robledo R.F., Glidden N.C., Hou G., Churchill G.A., Maser R.S. (2019). Genetic mapping in Diversity Outbred mice identifies a Trpa1 variant influencing late-phase formalin response. Pain.

[bib151] Reimann F., Cox J.J., Belfer I., Diatchenko L., Zaykin D.V., McHale D.P., Drenth J.P., Dai F., Wheeler J., Sanders F. (2010). Pain perception is altered by a nucleotide polymorphism in SCN9A. Proc. Natl. Acad. Sci. USA.

[bib152] Rice A.S., Cimino-Brown D., Eisenach J.C., Kontinen V.K., Lacroix-Fralish M.L., Machin I., Mogil J.S., Stöhr T., Preclinical Pain Consortium (2008). Animal models and the prediction of efficacy in clinical trials of analgesic drugs: a critical appraisal and call for uniform reporting standards. Pain.

[bib153] Rice A.S., Smith B.H., Blyth F.M. (2016). Pain and the global burden of disease. Pain.

[bib154] Rolke R., Magerl W., Campbell K.A., Schalber C., Caspari S., Birklein F., Treede R.D. (2006). Quantitative sensory testing: a comprehensive protocol for clinical trials. Eur. J. Pain.

[bib155] Rosen S.F., Ham B., Drouin S., Boachie N., Chabot-Dore A.J., Austin J.S., Diatchenko L., Mogil J.S. (2017). T-Cell Mediation of Pregnancy Analgesia Affecting Chronic Pain in Mice. J. Neurosci..

[bib156] Rosen S.F., Ham B., Haichin M., Walters I.C., Tohyama S., Sotocinal S.G., Mogil J.S. (2019). Increased pain sensitivity and decreased opioid analgesia in T-cell-deficient mice and implications for sex differences. Pain.

[bib157] Rowbotham M.C., Fields H.L. (1996). The relationship of pain, allodynia and thermal sensation in post-herpetic neuralgia. Brain.

[bib158] Russell W.M.S., Burch R.L., Hume C.W. (1959). The Principles of Humane Experimental Technique.

[bib159] Sabatowski R., Gálvez R., Cherry D.A., Jacquot F., Vincent E., Maisonobe P., Versavel M., Study G., 1008-045 Study Group (2004). Pregabalin reduces pain and improves sleep and mood disturbances in patients with post-herpetic neuralgia: results of a randomised, placebo-controlled clinical trial. Pain.

[bib160] Samuel G.N., Farsides B. (2017). The UK’s 100,000 Genomes Project: manifesting policymakers’ expectations. New Genet. Soc..

[bib161] Sato-Takeda M., Ihn H., Ohashi J., Tsuchiya N., Satake M., Arita H., Tamaki K., Hanaoka K., Tokunaga K., Yabe T. (2004). The human histocompatibility leukocyte antigen (HLA) haplotype is associated with the onset of postherpetic neuralgia after herpes zoster. Pain.

[bib162] Schrenk-Siemens K., Wende H., Prato V., Song K., Rostock C., Loewer A., Utikal J., Lewin G.R., Lechner S.G., Siemens J. (2015). PIEZO2 is required for mechanotransduction in human stem cell-derived touch receptors. Nat. Neurosci..

[bib163] Sekijima Y., Ueda M., Koike H., Misawa S., Ishii T., Ando Y. (2018). Diagnosis and management of transthyretin familial amyloid polyneuropathy in Japan: red-flag symptom clusters and treatment algorithm. Orphanet J. Rare Dis..

[bib164] Seltzer Z., Dubner R., Shir Y. (1990). A novel behavioral model of neuropathic pain disorders produced in rats by partial sciatic nerve injury. Pain.

[bib165] Shidahara Y., Ogawa S., Nakamura M., Nemoto S., Awaga Y., Takashima M., Hama A., Matsuda A., Takamatsu H. (2016). Pharmacological comparison of a nonhuman primate and a rat model of oxaliplatin-induced neuropathic cold hypersensitivity. Pharmacol. Res. Perspect..

[bib166] Shields S.D., Eckert W.A., Basbaum A.I. (2003). Spared nerve injury model of neuropathic pain in the mouse: a behavioral and anatomic analysis. J. Pain.

[bib167] Sieberg C.B., Taras C., Gomaa A., Nickerson C., Wong C., Ward C., Baskozos G., Bennett D.L.H., Ramirez J.D., Themistocleous A.C. (2018). Neuropathic pain drives anxiety behavior in mice, results consistent with anxiety levels in diabetic neuropathy patients. Pain Rep..

[bib168] Sittig L.J., Carbonetto P., Engel K.A., Krauss K.S., Barrios-Camacho C.M., Palmer A.A. (2016). Genetic Background Limits Generalizability of Genotype-Phenotype Relationships. Neuron.

[bib169] Smith E.S., Lewin G.R. (2009). Nociceptors: a phylogenetic view. J. Comp. Physiol. A Neuroethol. Sens. Neural Behav. Physiol..

[bib170] Sneddon L.U. (2018). Comparative Physiology of Nociception and Pain. Physiology (Bethesda).

[bib171] Sorge R.E., Mapplebeck J.C., Rosen S., Beggs S., Taves S., Alexander J.K., Martin L.J., Austin J.S., Sotocinal S.G., Chen D. (2015). Different immune cells mediate mechanical pain hypersensitivity in male and female mice. Nat. Neurosci..

[bib172] Strynatka K.A., Gurrola-Gal M.C., Berman J.N., McMaster C.R. (2018). How Surrogate and Chemical Genetics in Model Organisms Can Suggest Therapies for Human Genetic Diseases. Genetics.

[bib173] Sudlow C., Gallacher J., Allen N., Beral V., Burton P., Danesh J., Downey P., Elliott P., Green J., Landray M. (2015). UK biobank: an open access resource for identifying the causes of a wide range of complex diseases of middle and old age. PLoS Med..

[bib174] Sumiyama D., Kikkawa E.F., Kita Y.F., Shinagawa H., Mabuchi T., Ozawa A., Inoko H. (2008). HLA alleles are associated with postherpetic neuralgia but not with herpes zoster. Tokai J. Exp. Clin. Med..

[bib175] Tam V., Patel N., Turcotte M., Bossé Y., Paré G., Meyre D. (2019). Benefits and limitations of genome-wide association studies. Nat. Rev. Genet..

[bib176] Tannenbaum J., Bennett B.T. (2015). Russell and Burch’s 3Rs then and now: the need for clarity in definition and purpose. J. Am. Assoc. Lab. Anim. Sci..

[bib177] Tappe-Theodor A., King T., Morgan M.M. (2019). Pros and Cons of Clinically Relevant Methods to Assess Pain in Rodents. Neurosci. Biobehav. Rev..

[bib178] Taves S., Berta T., Liu D.L., Gan S., Chen G., Kim Y.H., Van de Ven T., Laufer S., Ji R.R. (2016). Spinal inhibition of p38 MAP kinase reduces inflammatory and neuropathic pain in male but not female mice: Sex-dependent microglial signaling in the spinal cord. Brain Behav. Immun..

[bib179] Tedeschi A., Omura T., Costigan M. (2017). CNS repair and axon regeneration: Using genetic variation to determine mechanisms. Exp. Neurol..

[bib180] Tegeder I., Costigan M., Griffin R.S., Abele A., Belfer I., Schmidt H., Ehnert C., Nejim J., Marian C., Scholz J. (2006). GTP cyclohydrolase and tetrahydrobiopterin regulate pain sensitivity and persistence. Nat. Med..

[bib181] Tracey W.D., Wilson R.I., Laurent G., Benzer S. (2003). painless, a Drosophila gene essential for nociception. Cell.

[bib182] Tracey I., Woolf C.J., Andrews N.A. (2019). Composite Pain Biomarker Signatures for Objective Assessment and Effective Treatment. Neuron.

[bib183] Tuttle A.H., Philip V.M., Chesler E.J., Mogil J.S. (2018). Comparing phenotypic variation between inbred and outbred mice. Nat. Methods.

[bib184] Upadhyay U., Zhuang G.Z., Diatchenko L., Parisien M., Kang Y., Sarantopoulos K.D., Martin E.R., Smith S.B., Maixner W., Levitt R.C. (2019). Profound analgesia is associated with a truncated peptide resulting from tissue specific alternative splicing of DRG CA8-204 regulated by an exon-level cis-eQTL. PLoS Genet..

[bib185] Usoskin D., Furlan A., Islam S., Abdo H., Lönnerberg P., Lou D., Hjerling-Leffler J., Haeggström J., Kharchenko O., Kharchenko P.V. (2015). Unbiased classification of sensory neuron types by large-scale single-cell RNA sequencing. Nat. Neurosci..

[bib186] van Hecke O., Austin S.K., Khan R.A., Smith B.H., Torrance N. (2014). Neuropathic pain in the general population: a systematic review of epidemiological studies. Pain.

[bib187] van Hecke O., Kamerman P.R., Attal N., Baron R., Bjornsdottir G., Bennett D.L., Bennett M.I., Bouhassira D., Diatchenko L., Freeman R. (2015). Neuropathic pain phenotyping by international consensus (NeuroPPIC) for genetic studies: a NeuPSIG systematic review, Delphi survey, and expert panel recommendations. Pain.

[bib188] Veluchamy A., Hébert H.L., Meng W., Palmer C.N.A., Smith B.H. (2018). Systematic review and meta-analysis of genetic risk factors for neuropathic pain. Pain.

[bib189] Volkow N.D., Koroshetz W.J. (2019). The role of neurologists in tackling the opioid epidemic. Nat. Rev. Neurol..

[bib190] Wakisaka S., Kajander K.C., Bennett G.J. (1991). Abnormal skin temperature and abnormal sympathetic vasomotor innervation in an experimental painful peripheral neuropathy. Pain.

[bib191] Waldron A.L., Schroder P.A., Bourgon K.L., Bolduc J.K., Miller J.L., Pellegrini A.D., Dubois A.L., Blaszkiewicz M., Townsend K.L., Rieger S. (2018). Oxidative stress-dependent MMP-13 activity underlies glucose neurotoxicity. J. Diabetes Complications.

[bib192] Walsh J., Rabey M.I., Hall T.M. (2012). Agreement and correlation between the self-report leeds assessment of neuropathic symptoms and signs and Douleur Neuropathique 4 Questions neuropathic pain screening tools in subjects with low back-related leg pain. J. Manipulative Physiol. Ther..

[bib193] Warner S.C., van Meurs J.B.J., Schiphof D., Bierma-Zeinstra S.M., Hofman A., Uitterlinden A.G., Richardson H., Jenkins W., Doherty M., Valdes A.M. (2017). Genome-wide association scan of neuropathic pain symptoms post total joint replacement highlights a variant in the protein-kinase C gene. Eur. J. Hum. Genet..

[bib194] Watanabe H., Numata K., Ito T., Takagi K., Matsukawa A. (2004). Innate immune response in Th1- and Th2-dominant mouse strains. Shock.

[bib195] Waterston R.H., Lindblad-Toh K., Birney E., Rogers J., Abril J.F., Agarwal P., Agarwala R., Ainscough R., Alexandersson M., An P., Mouse Genome Sequencing Consortium (2002). Initial sequencing and comparative analysis of the mouse genome. Nature.

[bib196] Weir G.A., Middleton S.J., Clark A.J., Daniel T., Khovanov N., McMahon S.B., Bennett D.L. (2017). Using an engineered glutamate-gated chloride channel to silence sensory neurons and treat neuropathic pain at the source. Brain.

[bib197] Wustmann G., Rein K., Wolf R., Heisenberg M. (1996). A new paradigm for operant conditioning of Drosophila melanogaster. J. Comp. Physiol. A Neuroethol. Sens. Neural Behav. Physiol..

[bib198] Xie W., Strong J.A., Zhang J.M. (2017). Active Nerve Regeneration with Failed Target Reinnervation Drives Persistent Neuropathic Pain. eNeuro.

[bib199] Yang Y., Wang Y., Li S., Xu Z., Li H., Ma L., Fan J., Bu D., Liu B., Fan Z. (2004). Mutations in SCN9A, encoding a sodium channel alpha subunit, in patients with primary erythermalgia. J. Med. Genet..

[bib200] Yoshiki A., Moriwaki K. (2006). Mouse phenome research: implications of genetic background. ILAR J..

[bib201] Zakrzewska J.M., Palmer J., Morisset V., Giblin G.M., Obermann M., Ettlin D.A., Cruccu G., Bendtsen L., Estacion M., Derjean D., study investigators (2017). Safety and efficacy of a Nav1.7 selective sodium channel blocker in patients with trigeminal neuralgia: a double-blind, placebo-controlled, randomised withdrawal phase 2a trial. Lancet Neurol..

[bib203] Zhang X., Priest B.T., Belfer I., Gold M.S. (2017). Voltage-gated Na^+^ currents in human dorsal root ganglion neurons. eLife.

[bib204] Zhang Y., Liu S., Zhang Y.Q., Goulding M., Wang Y.Q., Ma Q. (2018). Timing Mechanisms Underlying Gate Control by Feedforward Inhibition. Neuron.

[bib205] Zhao X., Tang Z., Zhang H., Atianjoh F.E., Zhao J.Y., Liang L., Wang W., Guan X., Kao S.C., Tiwari V. (2013). A long noncoding RNA contributes to neuropathic pain by silencing Kcna2 in primary afferent neurons. Nat. Neurosci..

[bib206] Zheng Y., Liu P., Bai L., Trimmer J.S., Bean B.P., Ginty D.D. (2019). Deep Sequencing of Somatosensory Neurons Reveals Molecular Determinants of Intrinsic Physiological Properties. Neuron.

[bib207] Zhuang G.Z., Upadhyay U., Tong X., Kang Y., Erasso D.M., Fu E.S., Sarantopoulos K.D., Martin E.R., Wiltshire T., Diatchenko L. (2018). Human carbonic anhydrase-8 AAV8 gene therapy inhibits nerve growth factor signaling producing prolonged analgesia and anti-hyperalgesia in mice. Gene Ther..

